# First-trimester preeclampsia prediction model via integrative machine learning of maternal risk profiles and laboratory markers

**DOI:** 10.1016/j.gendis.2026.102075

**Published:** 2026-02-09

**Authors:** Yi Zhu, Yanqiu Zhang, Chao Huang, Sheng Zhang, Jun Cao, Nicole Miranda, Sarina Zhao, Yan Peng, Chao Yu, Bin Feng, Jieyu Jin, Qingqin Tang, Jiaming Fan, Longwei Qiao, Yuting Liang

**Affiliations:** a Center for Clinical Laboratory, The First Affiliated Hospital of Soochow University, Suzhou, Jiangsu 215031, China; b Department of Orthopaedic Surgery, The First Affiliated Hospital of Soochow University, Suzhou, Jiangsu 215031, China; c Institute of Orthopaedics, Soochow University, Suzhou, Jiangsu 215000, China; d Molecular Oncology Laboratory, Department of Orthopedic Surgery and Rehabilitation Medicine, The University of Chicago Medical Center, Chicago, IL 60637, USA; e Institute of Clinical Pharmacology, School of Pharmacy, Anhui Medical University, Key Laboratory of Anti-inflammatory and Immune Medicine (Anhui Medical University), Ministry of Education, Hefei, Anhui 230032, China; f Center for Reproduction and Genetics, School of Gusu, The Affiliated Suzhou Hospital of Nanjing Medical University, Suzhou Municipal Hospital, Nanjing Medical University, Suzhou, Jiangsu 215002, China; g Departments of Orthopaedic Surgery, Cardiology, and Obstetrics and Gynecology, The Affiliated University-town Hospital, Chongqing Medical University, Chongqing 401331, China; h Ministry of Education Key Laboratory of Diagnostic Medicine, Department of Clinical Biochemistry, School of Laboratory Diagnostic Medicine, Chongqing Medical University, Chongqing 400016, China; i State Key Laboratory of Genetic Engineering, School of Life Sciences, Fudan University, Shanghai 200438, China

**Keywords:** FMF prediction model, Laboratory markers, Machine learning, Preeclampsia, Triglyceride-glucose index

## Abstract

Preeclampsia (PE) affects 2%–8% of pregnancies worldwide and remains a major cause of maternal and perinatal complications. Early risk identification is essential, yet current models, such as those from the Fetal Medicine Foundation (FMF), show limited sensitivity, especially for term PE. We investigated whether first-trimester routine laboratory markers could enhance PE prediction in a retrospective cohort of 12,715 pregnancies, including 556 (4.4%) PE cases. Elevated gamma-glutamyltransferase (GGT), C-reactive protein (CRP), triglyceride-glucose index multiple of the median (TyG MoM), and uric acid-to-albumin ratio multiple of the median (UAR MoM) were independently associated with a higher risk of PE, whereas magnesium, iron, and high-density lipoprotein cholesterol were inversely associated with the condition. Several markers also exhibited nonlinear and threshold effects. These features were incorporated into machine learning frameworks, with distinct models for preterm and term PE. For preterm PE, the CatBoost model achieved an AUC of 0.954 and detected 92.5% of cases at a 14% false-positive rate when integrated with the FMF model, compared with 65% detection with FMF alone. For term PE, logistic regression yielded an AUC of 0.913 and 81.1% detection, substantially outperforming FMF sensitivity (47.7%). Parallel cell-free DNA transcriptomic profiling revealed early metabolic and inflammatory dysregulation, and GEO dataset analyses provided external validation, supporting laboratory findings. These results indicate that the incorporation of accessible laboratory markers into existing clinical frameworks, combined with machine learning, provides a cost-effective and scalable strategy for enhanced PE risk stratification and precision management.

## Introduction

Preeclampsia (PE) is acknowledged as a significant contributor to both maternal and fetal mortality and is linked to a considerably heightened risk of long-term cardiovascular and chronic conditions after pregnancy, such as chronic hypertension, heart disease, stroke, metabolic syndrome, cognitive decline, and end-stage renal failure.^1^, ^2^, ^3^ Despite extensive research, the pathogenesis of PE remains unclear, and no effective treatment exists beyond delivery. Studies have shown that administering low-dose aspirin (50–150 mg/day) before 16 weeks of gestation can reduce the incidence of preterm PE by nearly 60%.^4^^,^^5^ Therefore, early identification of high-risk women is crucial for timely intervention and improved pregnancy outcomes.

The Fetal Medicine Foundation (FMF) models, which combine maternal factors, mean arterial pressure (MAP), uterine artery pulsatility index (UtA-PI), and placental growth factor (PLGF), are widely validated but have limited practicality due to complex assessments and specialized testing. Moreover, the FMF model detects approximately 75% of preterm PE cases (delivery before 37 weeks) and 40%–45% of term PE cases at a false-positive rate of 10%, leaving room for improvement.^6^ Routine laboratory markers assessing liver, kidney, lipid, glucose, and trace element profiles are standard in prenatal screening. PE is often accompanied by hepatic impairment, renal dysfunction, and metabolic disturbances, reflected in abnormalities such as elevated bilirubin, alanine aminotransferase (ALT), aspartate aminotransferase (AST), and uric acid (UA) levels. Yet, whether these markers exhibit predictive variability before PE onset remains understudied. Moreover, insulin resistance has also been identified as a key contributor to PE development.^7^ The triglyceride-glucose (TyG) index, derived from routine laboratory parameters, is a validated indicator of insulin resistance.^8^ Although well recognized in glucose metabolism disorders, its predictive value for early PE remains unclear.

To address these gaps, this study investigates whether first-trimester routine laboratory markers can predict PE. Initial analyses focus on identifying laboratory markers associated with PE and determining whether these relationships are linear or nonlinear. Where nonlinear associations are observed, threshold effects are further examined. Leveraging the power of machine learning capable of uncovering complex interactions between variables that traditional linear methods might miss, this study explores the predictive performance of multiple–indicator combinations within laboratory data. Additionally, it evaluates the integration of these findings with existing prediction models to assess potential improvements in predictive efficacy. This research aims to identify novel predictive markers for PE, offering a more accessible and accurate tool for early prevention.

## Materials and methods

### Study design and data collection

This retrospective cohort study included pregnant women who received antenatal care and delivered at Suzhou Municipal Hospital and the First Affiliated Hospital of Soochow University between 2015 and 2024. The Reproductive Medicine Ethics Committee of Suzhou Municipal Hospital reviewed and approved this research (K-2025-200-K01). All data were collected from the hospitals' electronic medical record systems. Laboratory markers test results collected before 14 weeks of gestation were considered as potential features for the development of the model for each participant. After excluding 140 cases of fetal demise, termination, or spontaneous abortion, 12,715 pregnancies remained for analysis. Given the retrospective nature of the study, informed consent was waived by the institutional review board. All patient information was anonymized in accordance with the Declaration of Helsinki.

### Laboratory parameter measurement and statistical analysis

Blood samples for laboratory markers testing were collected during the routine first-trimester screening visit, scheduled between 11 and 13^+6^ weeks of gestation. The median gestational age at blood sampling for the entire cohort was 12.43 weeks (interquartile range: 11.81–13.10 weeks). In the control group, the median was 12.43 weeks (interquartile range: 11.86–13.10 weeks), while in the PE group, it was 12.29 weeks (interquartile range: 11.71–13.00 weeks); the difference between groups was not statistically significant (*p* = 0.283) (Table S1).

Data collected included demographic characteristics, laboratory markers test results, and ultrasound screening parameters, all obtained within the same early pregnancy clinical assessment protocol (11–13^+6^ weeks). Clinical variables included MAP, maternal age, weight, height, gravidity, parity, plurality (*e.g.*, twin *vs*. singleton gestation), and mode of conception (natural *vs*. assisted reproduction–*in vitro* fertilization/IVF). Additional maternal history variables included smoking status, history of hypertension, diabetes, fetal growth restriction, PE, obstetric complications, and medication use during pregnancy. Laboratory markers included the angiogenic marker PLGF; liver function indices: total bile acids (TBA), albumin-to-globulin ratio (A/G), total bilirubin (TBIL), direct bilirubin (DBIL), indirect bilirubin (IBIL), prealbumin (PALB), globulin (GLB), ALT, alkaline phosphatase (ALP), gamma-glutamyltransferase (GGT), lactate dehydrogenase (LDH), and cholinesterase (CHE); metabolic and renal markers: fasting blood glucose (FBG), UA, blood urea, and creatinine (Cr); electrolytes and minerals: total carbon dioxide (CO_2_), sodium (Na), potassium (K), calcium (Ca), magnesium (Mg), copper (Cu), iron (Fe), chloride (Cl), phosphorus (P), and zinc (Zn); lipid profile: triglycerides (TG), total cholesterol (TC), high-density lipoprotein cholesterol (HDL-C), low-density lipoprotein cholesterol (LDL-C), and very low-density lipoprotein cholesterol (VLDL-C); inflammation marker C-reactive protein (CRP); derived indices: triglyceride-glucose index multiple of the median (TyG MoM) and uric acid-to-albumin ratio multiple of the median (UAR MoM); other markers: total protein (TP), albumin (ALB), AST, hydroxybutyrate dehydrogenase (HBDH), creatine kinase (CK); ultrasound parameters: ultrasound data included the UtA-PI measured during first-trimester screening. Pregnancy outcomes and complications: Information on pregnancy outcomes and obstetric complications was collected from hospital medical records and discharge summaries. Furthermore, missing laboratory or clinical data were imputed using the median value calculated from the available data for each respective variable. This approach ensures that imputed values align with the overall data distribution and helps mitigate potential biases due to missing data. Variables with a missing rate exceeding 20% were excluded from the analysis.

The primary outcome of this study was the development of PE, defined as new-onset hypertension accompanied by proteinuria. Hypertension was defined as a systolic blood pressure ≥ 140 mmHg and/or a diastolic blood pressure ≥ 90 mmHg. Proteinuria was defined by any of the following criteria: at least two readings of ≥ 1+ on a urine dipstick test, ≥ 300 mg of protein in a 24-h urine collection, or a protein-to-creatinine ratio ≥ 30 mg/mmol. In the absence of proteinuria, a diagnosis of PE could also be made if any of the following severe features were present: thrombocytopenia (platelet count < 100 × 10^9^/L), impaired liver function (serum transaminases > 2 times the upper limit of normal), renal insufficiency (serum creatinine > 1.1 mg/dL or > 2 times baseline), pulmonary edema, or new-onset neurological symptoms including visual disturbances. PE was further categorized based on gestational age at delivery into preterm PE (< 37 weeks of gestation) and term PE (≥ 37 weeks of gestation).

The baseline characteristics of the study participants were presented as the median (first quartile, third quartile) for continuous variables and the number (percentage) for categorical variables. The Mann–Whitney *U* test and Chi-square test were used to assess differences between groups for continuous and categorical variables, respectively. Multivariate logistic regression models (Model 1, Model 2, and Model 3) were employed to evaluate the association between maternal early laboratory markers and PE. Model 1 (Crude model) did not adjust for covariates, Model 2 adjusted for maternal age, body mass index (BMI), and plurality, and Model 3 adjusted for maternal age, BMI, plurality, parity, and IVF conception. Additional trend analyses were also conducted. Laboratory markers were first divided into quartiles, and the medians of each group were then introduced as continuous variables into the logistic regression model to identify potential linear trends in the association. Results were expressed as odds ratios (OR) and 95% confidence intervals (CI). Additionally, we conducted restricted cubic spline (RCS) analysis to assess the nonlinear relationship between laboratory markers and the risk of PE, using likelihood ratio tests to detect nonlinearity. The threshold effects of laboratory markers on PE risk were further analyzed using a two-stage linear regression model.

### Machine learning model development and validation

A total of 12,715 pregnant women were included, comprising 12,159 without PE, 262 with preterm PE, and 294 with term PE. Two analyses were performed: one for preterm PE (12,421 women: 262 cases and 12,159 controls) and one for term PE (12,453 women: 294 cases and 12,159 controls). Each dataset was randomly split into training and testing sets (8:2). Feature selection was performed on the training set using the Boruta algorithm based on the random forest classifier. This algorithm identifies relevant features by comparing the importance scores of actual variables with those of shadow features, which are randomly permuted versions of the original variables. As a result, 22 out of 26 differentially expressed features were deemed important and selected for preterm PE, and 19 out of 26 for term PE. These features were incorporated into seven different machine learning models: logistic regression, random forest, k-nearest neighbors (KNN), adaptive boosting (AdaBoost), categorical boosting (CatBoost), extreme gradient boosting (XGBoost), and light gradient boosting machine (LightGBM).

For each model, hyperparameters were optimized using Bayesian optimization to maximize the area under the receiver operating characteristic (ROC) curve (AUC) on the training set. Ten-fold cross-validation was employed to ensure robustness and reliability. ROC curves and sensitivity values were calculated on the testing set to evaluate model performance. The model with the best combination of AUC and sensitivity was selected as the optimal model. To enhance interpretability, SHapley Additive exPlanations (SHAP) were computed to quantify the contribution of each feature to the model's predictions, and SHAP summary plots were generated to visualize feature impacts.

Among the cohort, 5901 pregnant women underwent PE risk assessment using the FMF clinical model, which incorporated maternal risk factors, MAP, PLGF, and UtA-PI. The study population included 80 cases of preterm PE, 153 cases of term PE, and 5668 controls without PE. For model development, two analyses were conducted: one for preterm PE (80 cases and 5668 controls) and one for term PE (153 cases and 5668 controls). The datasets were randomly divided into training and testing sets in an 8:2 ratio. Input variables included maternal risk factors, MAP, PLGF, UtA-PI, and prediction scores from the preliminary models (AdaBoost for preterm PE and LightGBM for term PE). Seven machine learning algorithms were evaluated, and the optimal model was selected based on the AUC and sensitivity.

### Cell-free DNA sequencing and gene function analysis

Maternal plasma samples were collected during mid-pregnancy, before any clinical diagnosis of PE, from 51 pregnant individuals undergoing routine first-trimester screening. Of these, 18 individuals were later diagnosed with preterm PE, defined as PE requiring delivery before 37 weeks of gestation, based on criteria from the American College of Obstetricians and Gynecologists (ACOG).

Approximately 5 mL of maternal peripheral blood was drawn into EDTA-containing tubes. Plasma was isolated by sequential centrifugation at 1600 *g* for 10 min and 16,000 *g* for 10 min at 4 °C. Plasma aliquots were carefully transferred to fresh Eppendorf tubes and stored at −80 °C until processing.

Cell-free DNA (cfDNA) was extracted from 200 μL of plasma using a commercial Circulating DNA from Plasma Kit (GenMag Biotech, Beijing, China), following the manufacturer's instructions. Sequencing libraries were prepared according to the manufacturer's protocols. Library concentrations were measured using a Qubit 2.0 fluorometer (Invitrogen, Carlsbad, California, USA), and fragment size distribution was verified using the Agilent High Sensitivity DNA Kit and a 2100 Bioanalyzer (Agilent Technologies, Palo Alto, California, USA). Indexed libraries were pooled and sequenced on the MGISEQ-2000 platform using paired-end 100 bp reads. A minimum of 128 million reads was generated per sample.

Sequencing reads were aligned to the human reference genome (hg19) using BWA-MEM.^9^^,^^10^ PCR duplicates were removed using the MarkDuplicates function from Picard tools (version 1.119). Sequencing depth and coverage were assessed using the mpileup function of samtools. Genomic annotations were retrieved from RefSeq (University of California, Santa Cruz).^11^

To quantify promoter coverage, the ±1 kb region flanking the transcription start site (TSS) for each gene was defined as the promoter region. Read counts were computed in 10 bp bins using bamCoverage to generate bigWig files. The computeMatrix function was used to extract read coverage across each promoter region. To correct for variations in sequencing depth across samples, raw coverage values were normalized using a modified reads-per-million method according to the formula: promoter proofing of TSSi = coverage of TSSi proofing ÷ coverage of total TSSi proofing × 10^6^.

Normalized TSS coverage profiles were compared between preterm PE cases and controls. Genes with significant differential coverage were identified using the Mann–Whitney *U* test, and Benjamini-Hochberg correction was applied to control the false discovery rate. Gene set enrichment analysis (GSEA) was then performed using the fgsea R package with Hallmark, Kyoto Encyclopedia of Genes and Genomes (KEGG), Reactome, and WikiPathways gene sets sourced from the Molecular Signatures database (MSigDB). Enrichment scores were calculated for each pathway, and significance was determined based on normalized enrichment score (NES), nominal *p* value, and adjusted false discovery rate < 0.25.

### Acquisition of the GEO datasets and differential expression analysis

Three placental tissue-derived gene expression microarray datasets (GSE10588,^12^ GSE25906,^13^ and GSE75010^14^) were retrieved from the GEO database (http://www.ncbi.nlm.nih.gov/geo/) according to the following criteria: sample size ≥ 5 per group (PE *vs*. controls); clinically confirmed diagnosis of PE based on ACOG criteria; exclusive use of placental tissue samples (excluding cell lines or other tissue). GSE10588, based on the GPL2986 platform, comprised 26 control and 17 PE samples; GSE25906, based on GPL6102, included 37 controls and 23 PE samples; and GSE75010, based on GPL6244, contained 77 controls and 80 PE samples. Data preprocessing, including standardization, probe annotation, and normalization, was performed using the limma R package. The three datasets were combined to form a unified expression matrix. Given that the chip data was measured by different platforms, the sva R package was applied to remove batch effects, and principal component analysis was performed to evaluate sample distribution before and after correction. Differentially expressed genes (DEGs) between PE and control groups were identified using the limma package, applying thresholds of |log2 fold change| > 0.2 and adjusted *p* value (*P*.adj) < 0.05. Volcano plots were generated with the ggplot2 package to visualize expression patterns. Subsequently, the identified DEGs were intersected with mid-pregnancy cfDNA-derived DEGs (*P*.adj < 0.05), with results visualized through a Venn diagram.

## Results

### Characteristics of study population

**Figure 1:**
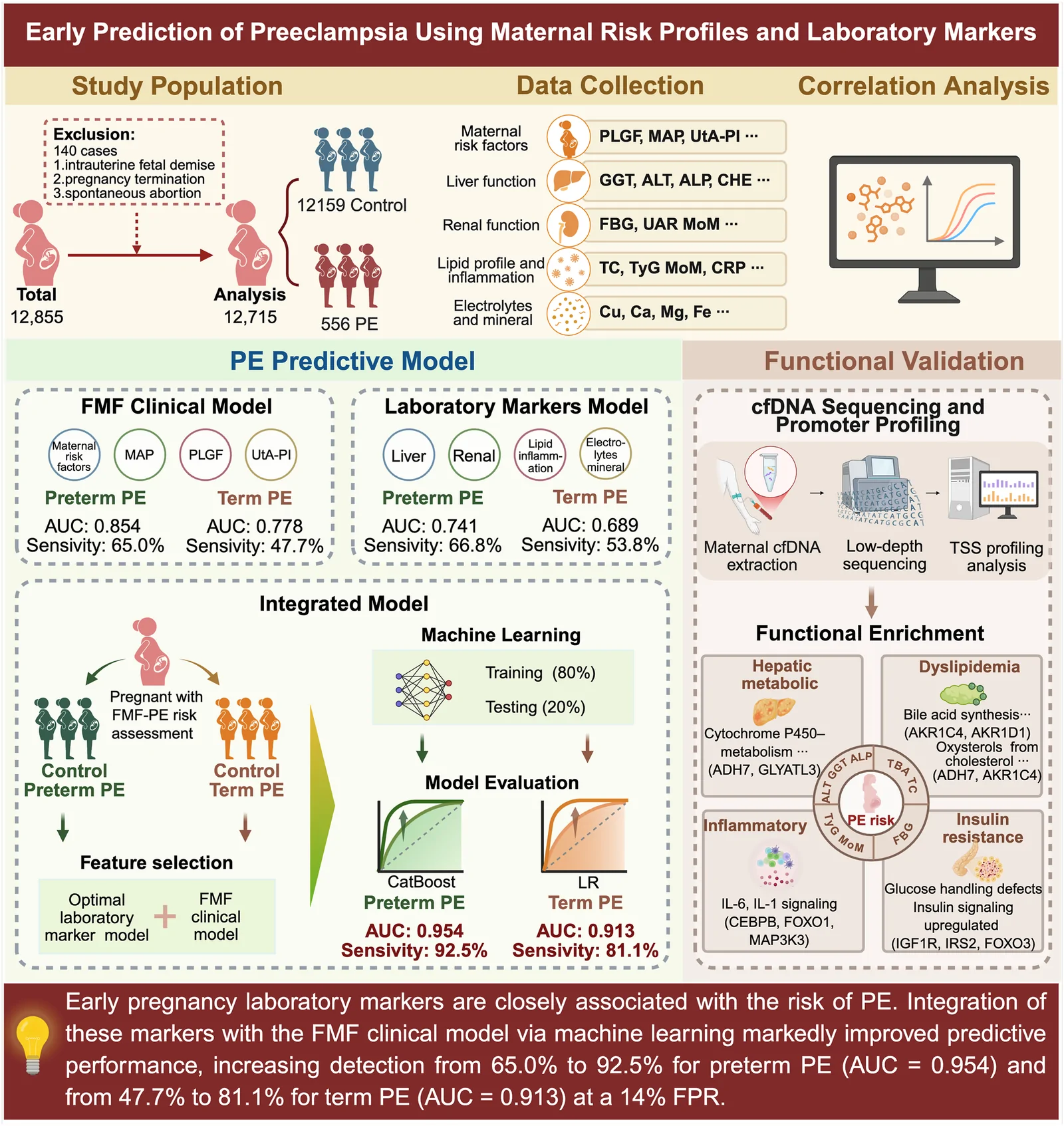
Flowchart of the study. This study included 12,715 pregnancies (with 140 cases of abnormal pregnancy excluded), among which 556 cases developed PE. Clinical data (age, BMI, plurality, parity, IVF, etc.), ultrasound parameters (UtA-PI), and laboratory markers (liver function, blood lipids, inflammation, trace elements, etc.) were collected before 14 weeks of gestation. Multivariate logistic regression, RCS analysis, and two-stage linear regression models were used to evaluate the association and threshold effects between laboratory markers and PE. Subsequently, the preterm PE and term PE cohorts were randomly divided into training and testing sets at an 8:2 ratio, respectively. The Boruta algorithm and seven machine learning models were employed for feature selection and the construction of laboratory markers prediction models. The optimal model was selected based on AUC and sensitivity, and SHAP values were used to quantify the contribution of features. Next, the prediction score of the optimal laboratory markers prediction model was integrated with the FMF model (maternal risk factors, MAP, PLGF, and UtA-PI) for the construction of the predictive modeling of PE, and the prediction performance was evaluated. Finally, cfDNA sequencing and TSS profiling were performed on 51 maternal plasma samples (18 cases of preterm PE and 33 controls), revealing early metabolic dysfunction and heightened inflammation in cases destined for PE. PE, preeclampsia; BMI, body mass index; IVF, *in vitro* fertilization; UtA-PI, uterine artery pulsatility index; RCS, restricted cubic spline; AUC, area under the receiver operating characteristic curve; SHAP, shapley additive explanations; FMF, fetal medicine foundation; MAP, mean arterial pressure; PLGF, placental growth factor; cfDNA, cell-free DNA; TSS, transcription start site; GGT, gamma-glutamyltransferase; ALT, alanine aminotransferase; ALP, alkaline phosphatase; CHE, cholinesterase; FBG, fasting blood glucose; UAR MoM, uric acid-albumin ratio multiple of the median; TC, total cholesterol; TyG MoM, triglyceride-glucose index multiple of the median; CRP, c-reactive protein; Cu, copper; Ca, calcium; Mg, magnesium; Fe, iron; CatBoost, categorical boosting; LR, logistic regression.

**Figure 2:**
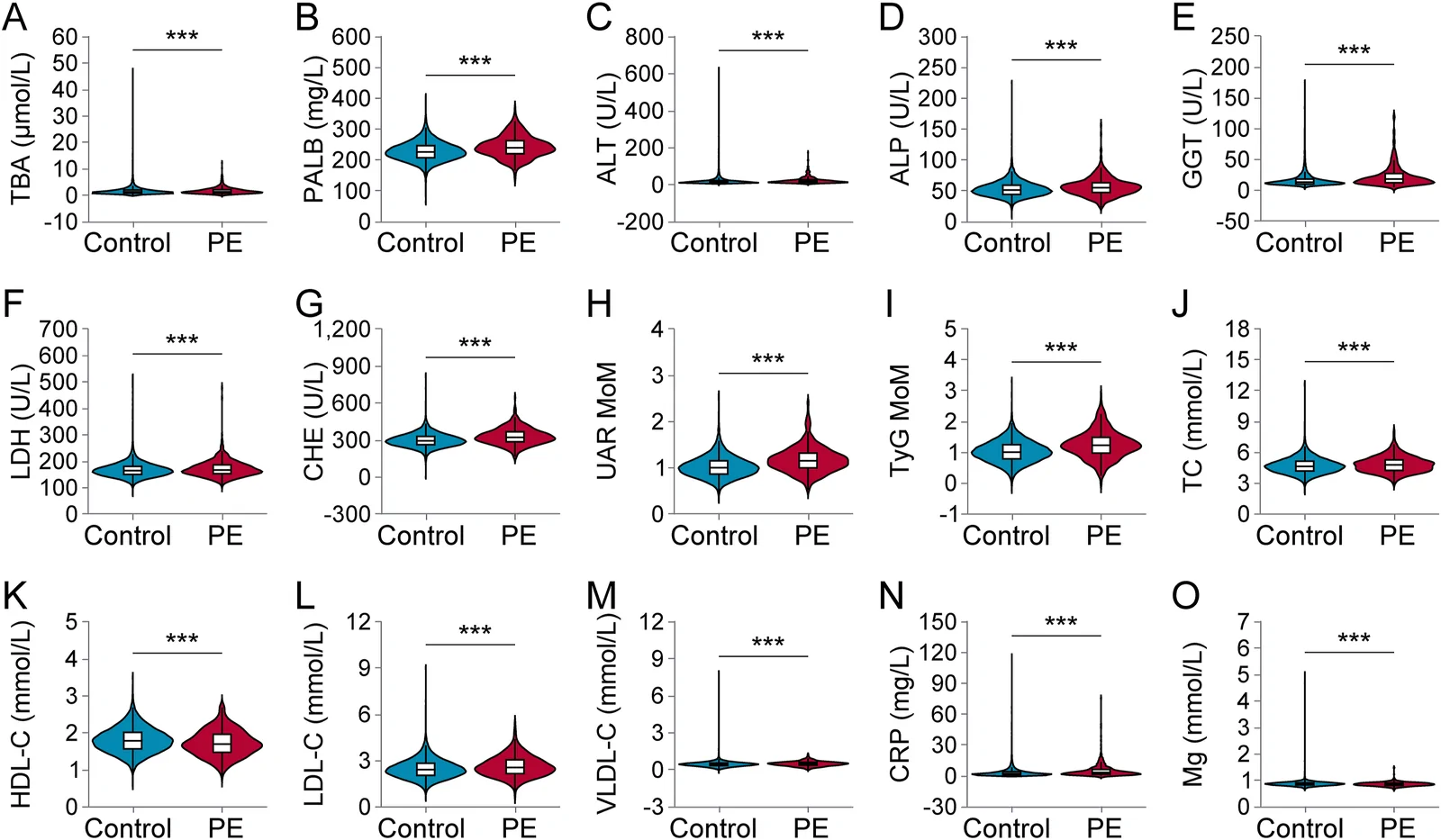
Laboratory markers changes in early pregnancy in PE pregnant women. Differences in **(A)** TBA, **(B)** PALB, **(C)** ALT, **(D)** ALP, **(E)** GGT, **(F)** LDH, **(G)** CHE, **(H)** UAR MoM, **(I)** TyG MoM, **(J)** TC, **(K)** HDL-C, **(L)** LDL-C, **(M)** VLDL-C, **(N)** CRP, and **(O)** Mg levels between PE cases and controls. PE, preeclampsia; TBA, total bile acids; PALB, prealbumin; ALT, alanine aminotransferase; ALP, alkaline phosphatase; GGT, gamma-glutamyltransferase; LDH, lactate dehydrogenase; CHE, cholinesterase; UAR MoM, uric acid-albumin ratio multiple of the median; TyG MoM, triglyceride-glucose index multiple of the median; TC, total cholesterol; HDL-C, high-density lipoprotein cholesterol; LDL-C, low-density lipoprotein cholesterol; VLDL-C, very low-density lipoprotein cholesterol; CRP, C-reactive protein; Mg, magnesium. ∗∗∗*p* < 0.001.

In this study, a comprehensive analytical framework was established to evaluate the predictive value of first-trimester laboratory markers for PE, with the overall study design and workflow summarized in Figure 1. Of the 12,715 pregnancies in the study population, PE occurred in 556 cases (4.4%). The characteristics of the study population are summarized in Figure 2 and Table S1. Compared with the non-PE group, the PE group exhibited higher maternal age and BMI and a higher prevalence of twin gestation and IVF conception, along with a lower parity. Before the 14th week of gestation, the PE group showed significantly higher levels of liver function-related indices, including TBA, PALB, GLB, ALT, ALP, GGT, LDH, and CHE, and lower levels of A/G, TBIL, DBIL, and IBIL. Additionally, renal function-related indices such as the UAR MoM, along with lipid- and inflammation-related indices, such as TyG MoM, TC, LDL-C, VLDL-C, and CRP, were significantly elevated in the PE group. In contrast, HDL-C levels were decreased in the PE group. Furthermore, micronutrient-related indices such as Na, K, Ca, and Cu were elevated, while CO_2_, Mg, and Fe levels were reduced in the PE group.

### Association analysis of routine laboratory markers and risk of PE

**Figure 3:**
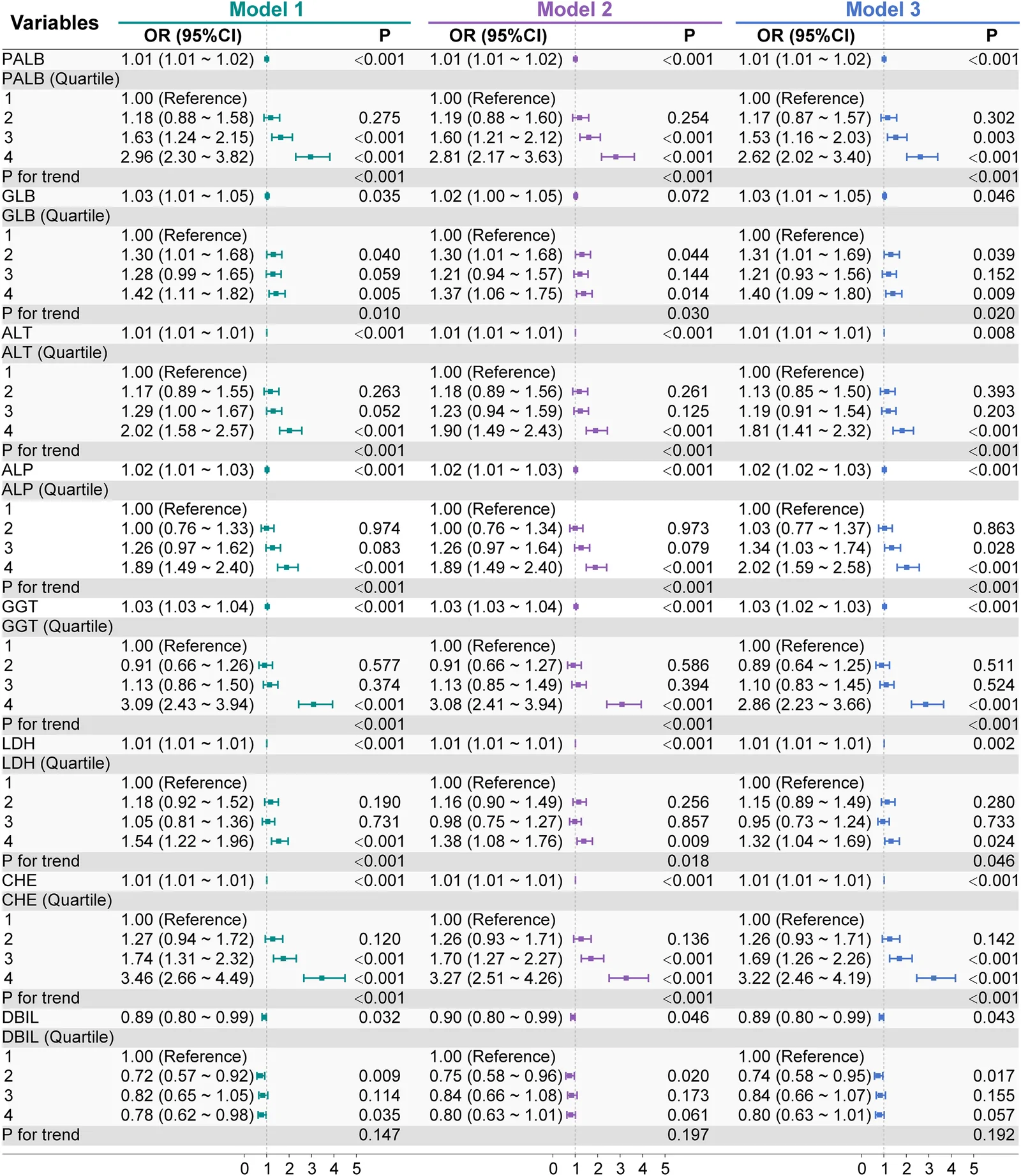
Association between liver function-related indices in early pregnancy and the risk of PE. Model 1: unadjusted model; Model 2: adjusted for age, BMI, and plurality; Model 3: adjusted for age, BMI, plurality, parity, and IVF conception. PE, preeclampsia; BMI, body mass index; IVF, *in vitro* fertilization; OR, odds ratio; CI, confidence interval; PALB, prealbumin; GLB, globulin; ALT, alanine aminotransferase; ALP, alkaline phosphatase; GGT, gamma-glutamyltransferase; LDH, lactate dehydrogenase; CHE, cholinesterase; DBIL, direct bilirubin.

In order to exclude the interference of confounding factors and reveal the true independent associations between various laboratory markers and PE risk, the study used three models to gradually adjust for potential confounding variables (Fig. 3; Table S2). In Model 3, which included adjustments for all confounding variables, liver function-related indices, including PALB (OR = 1.01, 95% CI, 1.01–1.02, *p* < 0.001), GLB (OR = 1.03, 95% CI, 1.01–1.05, *p* = 0.046), ALT (OR = 1.01, 95% CI, 1.01–1.01, *p* = 0.008), ALP (OR = 1.02, 95% CI, 1.02–1.03, *p* < 0.001), GGT (OR = 1.03, 95% CI, 1.02–1.03, *p* < 0.001), LDH (OR = 1.01, 95% CI, 1.01–1.01, *p* = 0.002), and CHE (OR = 1.01, 95% CI, 1.01–1.01, *p* < 0.001), were found to be significantly positively associated with PE prevalence, and DBIL (OR = 0.89, 95% CI, 0.80–0.99, *p* = 0.043) was significantly negatively associated with PE risk. Similarly, renal function-related indices, such as the UAR MoM (OR = 10.16, 95% CI, 7.33–14.09, *p* < 0.001), as well as lipid- and inflammation-related indices, including TyG MoM (OR = 4.29, 95% CI, 3.40–5.42, *p* < 0.001), TC (OR = 1.17, 95% CI, 1.05–1.30, *p* = 0.005), LDL-C (OR = 1.39, 95% CI, 1.22–1.57, *p* < 0.001), VLDL-C (OR = 1.86, 95% CI, 1.25–2.77, *p* = 0.002), and CRP (OR = 1.05, 95% CI, 1.03–1.06, *p* < 0.001), were also positively associated with PE prevalence. In contrast, HDL-C levels (OR = 0.48, 95% CI, 0.37–0.63, *p* < 0.001) were significantly inversely associated with PE risk. Additionally, micronutrient-related indices, such as Ca (OR = 4.03, 95% CI, 1.86–8.72, *p* < 0.001) and Cu (OR = 1.03, 95% CI, 1.01–1.05, *p* = 0.001), were positively associated with PE prevalence, whereas CO_2_ (OR = 0.96, 95% CI, 0.93–0.99, *p* = 0.021), Mg (OR = 0.07, 95% CI, 0.02–0.27, *p* < 0.001), and Fe (OR = 0.97, 95% CI, 0.96–0.99, *p* < 0.001) levels were inversely associated with PE risk. However, TBA, A/G, TBIL, IBIL, Na, and K were not found to be significantly associated with PE risk.

### Quartile analysis reveals dose-effect trends

**Figure 4:**
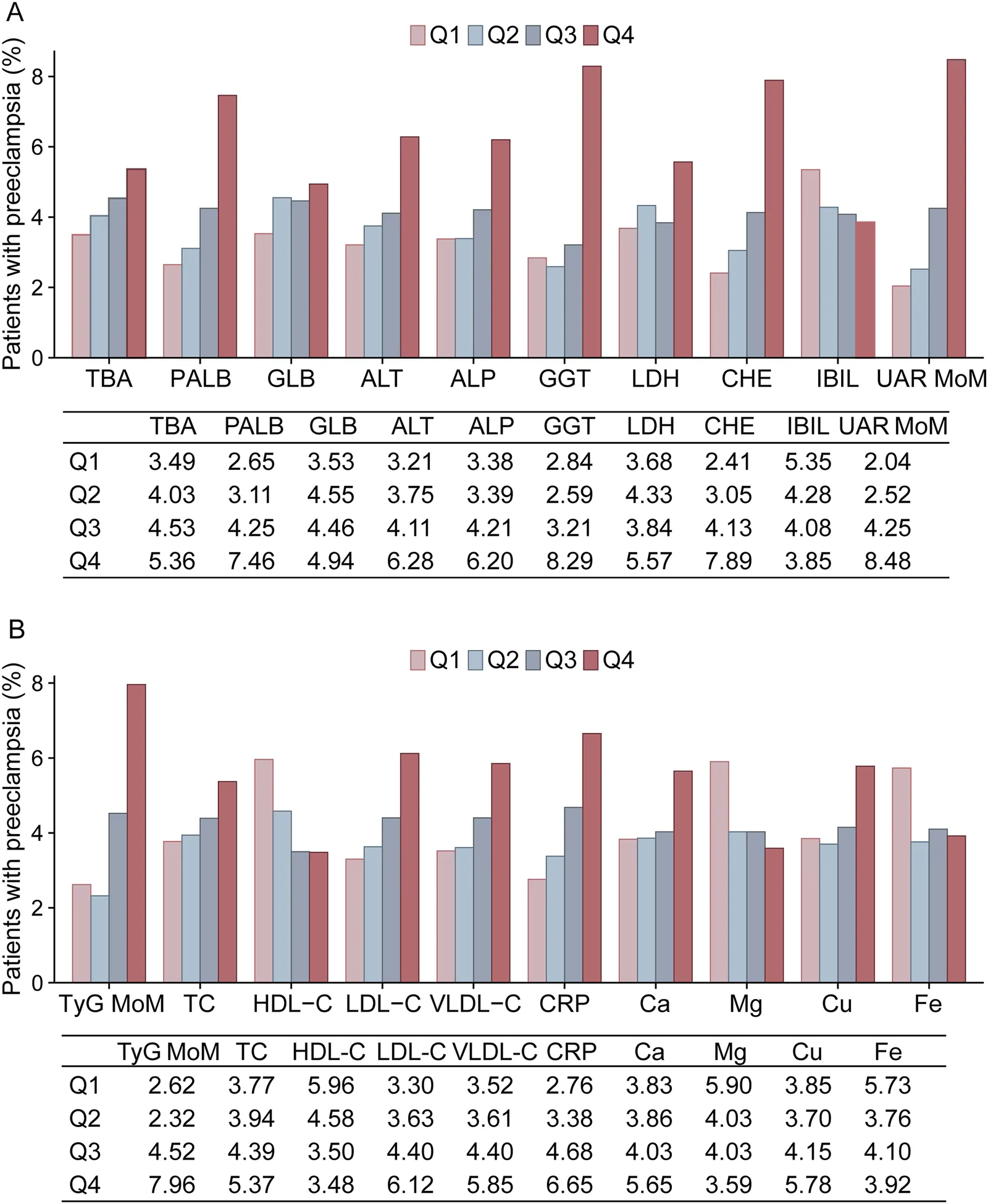
The proportion of patients with PE sorted by quartiles. **(A)** The proportion of patients with PE sorted by quartiles of liver function-related indices and renal function-related indicators (TBA, PALB, GLB, ALT, ALP, GGT, LDH, CHE, IBIL, and UAR MoM). **(B)** The proportion of patients with PE sorted by quartiles of lipid-, inflammation- and micronutrient-related indicators (TyG MoM, TC, HDL-C, LDL-C, VLDL-C, CRP, Ca, Mg, Cu, and Fe). PE, preeclampsia; TBA, total bile acids; PALB, prealbumin; GLB, globulin; ALT, alanine aminotransferase; ALP, alkaline phosphatase; GGT, gamma-glutamyltransferase; LDH, lactate dehydrogenase; CHE, cholinesterase; IBIL, indirect bilirubin; UAR MoM, uric acid-albumin ratio multiple of the median; TyG MoM, triglyceride-glucose index multiple of the median; TC, total cholesterol; HDL-C, high-density lipoprotein cholesterol; LDL-C, low-density lipoprotein cholesterol; VLDL-C, very low-density lipoprotein cholesterol; CRP, c-reactive protein; Ca, calcium; Mg, magnesium; Cu, copper; Fe, iron.

To investigate the potential nonlinear relationship between laboratory routine indicators and PE, as well as to identify high-risk populations, continuous laboratory markers were categorized into quartiles. As shown in Figure 3, Figure 4A and Table S2, when compared with the first quartile, a significantly higher prevalence of PE was observed in the fourth quartile of liver function-related indices, including TBA, PALB, GLB, ALT, ALP, GGT, LDH, and CHE, with increases of 0.54, 1.82, 0.4, 0.96, 0.83, 1.92, 0.51, and 2.27, respectively. However, the prevalence of PE was lower in the fourth quartile of IBIL levels.

Similarly, PE prevalence was significantly higher in the fourth quartile of renal function-related indices, such as UAR MoM (Fig. 4A), as well as in lipid- and inflammation-related indices, including TyG MoM, TC, LDL-C, VLDL-C, and CRP (Fig. 4B). Conversely, a significantly lower incidence of PE was found in the highest quartile of HDL-C (Fig. 4B). Additionally, an increased incidence of PE was noted in the fourth quartile of micronutrient-related indices, such as Ca and Cu, whereas a reduction in PE incidence was observed in the fourth quartile of Mg and Fe (Fig. 4B). This trend remained significant after adjusting for potential confounders (Table S2).

### Exploring the nonlinear relationship and threshold effects of laboratory markers in preeclampsia risk

**Figure 5:**
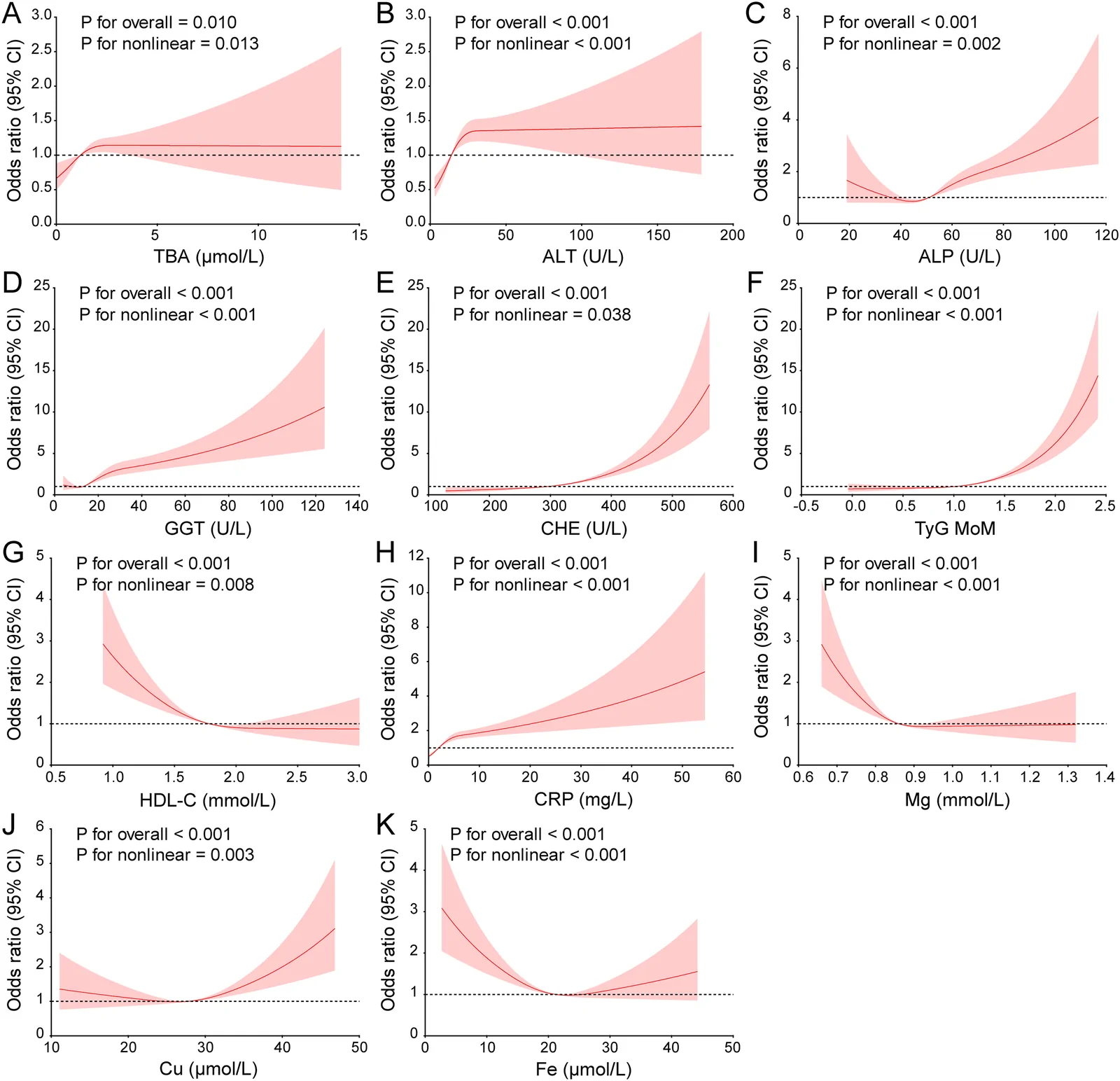
The nonlinear relationship between laboratory markers and PE risk. Restricted cubic spline analyses the association of PE with laboratory markers: **(A)** TBA; **(B)** ALT; **(C)** ALP; **(D)** GGT; **(E)** CHE; **(F)** TyG MoM; **(G)** HDL-C; **(H)** CRP; **(I)** Mg; **(J)** Cu; **(K)** Fe. PE, preeclampsia; TBA, total bile acids; ALT, alanine aminotransferase; ALP, alkaline phosphatase; GGT, gamma-glutamyltransferase; CHE, cholinesterase; TyG MoM, triglyceride-glucose index multiple of the median; HDL-C, high-density lipoprotein cholesterol; CRP, c-reactive protein; Mg, magnesium; Cu, copper; Fe, iron.

To further ensure the reliability of the findings and identify potential threshold effects between laboratory routine indicators and PE risk, a nonlinear relationship analysis was conducted using an RCS regression model. After adjusting for all confounders, nonlinear associations with PE risk were observed for TBA, ALT, ALP, GGT, CHE, TyG MoM, HDL-C, CRP, Mg, Cu, and Fe (*P* for nonlinearity < 0.05) (Fig. 5). In contrast, linear relationships with PE risk were identified for A/G, PALB, GLB, LDH, TBIL, DBIL, IBIL, UAR MoM, TC, LDL-C, VLDL-C, Na, K, Ca, and CO_2_ (*P* for nonlinearity > 0.05).

Subsequently, the threshold effects of laboratory routine indicators were examined to assess potential non-linear associations with the risk of PE. As shown in Table S3, threshold effects were identified for TBA, ALT, GGT, and CRP, with respective thresholds of 2.59 μmol/L, 37 U/L, 35 U/L, and 8.09 mg/L. Below these thresholds, each one-unit increase in the corresponding marker was associated with an elevated risk of PE by 0.24-fold (OR = 1.24; 95% CI, 1.05–1.45; *p* = 0.009), 0.03-fold (OR = 1.03; 95% CI, 1.02–1.04; *p* < 0.001), 0.06-fold (OR = 1.06; 95% CI, 1.04–1.07; *p* < 0.001), and 0.18-fold (OR = 1.18; 95% CI, 1.13–1.24; *p* < 0.001), respectively. However, no significant associations were observed above these thresholds. Similarly, threshold values were determined for ALP (41 U/L), CHE (254 U/L), TyG MoM (0.83), and Cu (31.39 μmol/L). Below these cut-off points, no significant associations with PE risk were observed. In contrast, above these cut-off values, each one-unit increase in the respective indicators was associated with increased PE risk by 0.03-fold (OR = 1.03; 95% CI, 1.02–1.03; *p* < 0.001), 0.01-fold (OR = 1.01; 95% CI, 1.01–1.01; *p* < 0.001), 5.06-fold (OR = 6.06; 95% CI, 4.50–8.17; *p* < 0.001), and 0.09-fold (OR = 1.09; 95% CI, 1.04–1.14; *p* < 0.001), respectively. For HDL-C, Mg, and Fe, the threshold values were 1.84 mmol/L, 0.91 mmol/L, and 25.1 μmol/L, respectively. Below these thresholds, each one-unit increase was associated with a reduced risk of PE by 0.70-fold (OR = 0.30; 95% CI, 0.18–0.51; *p* < 0.001), 0.98-fold (OR = 0.02; 95% CI, 0.00–0.13; *p* < 0.001), and 0.05-fold (OR = 0.95; 95% CI, 0.93–0.97; *p* < 0.001), respectively. No significant associations were identified above these cut-off thresholds.

### Laboratory markers predict pre-symptomatic PE

Based on these findings, the study further utilized machine learning to evaluate the predictive efficacy of these laboratory indicators. As previous studies have indicated that FMF models vary in predictive efficacy for different subtypes of PE, such as preterm and term PE, we sought to determine whether routine laboratory markers measured in early pregnancy could predict these PE subtypes.

**Figure 6:**
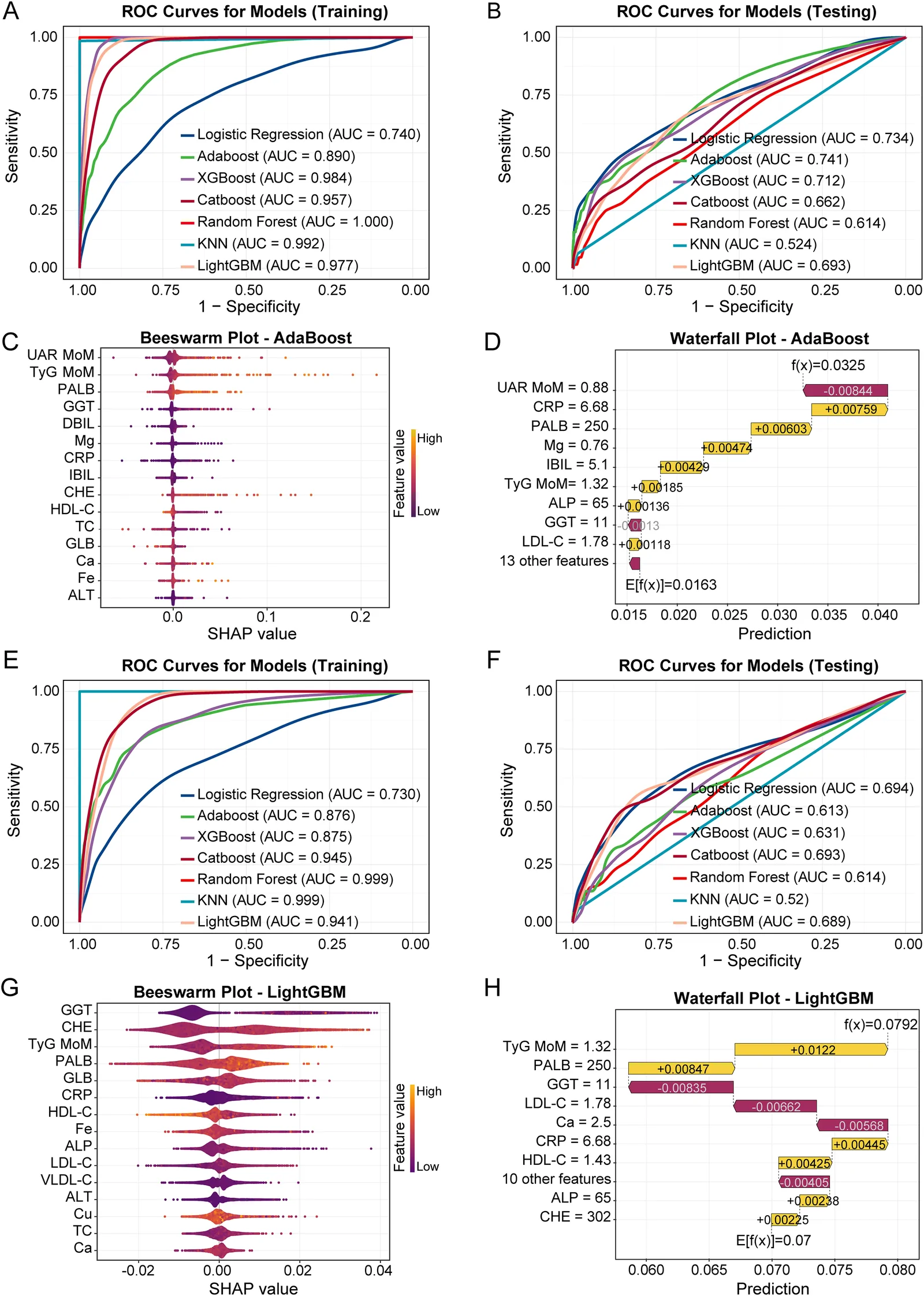
Laboratory markers predict pre-symptomatic PE. **(A)** The ROC curves of the preterm PE prediction models constructed by seven machine learning algorithms in the training set. **(B)** The ROC curves of the preterm PE prediction models constructed by seven machine learning algorithms in the testing set. **(C)** The SHAP summary plot of the AdaBoost model shows the effect of laboratory markers on the preterm PE prediction model. **(D)** Waterfall plot demonstrates the impact of specific laboratory markers on the predicted risk of preterm PE. **(E)** The ROC curves of the term PE prediction models constructed by seven machine learning algorithms in the training set. **(F)** The ROC curves of the term PE prediction models constructed by seven machine learning algorithms in the testing set. **(G)** The SHAP summary plot of the LightGBM model shows the effect of laboratory markers on the term PE prediction model. **(H)** Waterfall plot demonstrates the impact of specific laboratory markers on the predicted risk of term PE. PE, preeclampsia; ROC, receiver operating characteristic; SHAP, shapley additive explanations.

For preterm PE, 22 out of 26 differentially expressed features were identified as significant by the Boruta algorithm and were selected for model development. These 22 features were input into seven machine learning models, and the optimal combination of hyperparameters for preterm PE prediction was determined through 10-fold cross-validation. Model performance was assessed using sensitivity, AUC, and F1 score on the testing set. The AdaBoost model demonstrated both the highest AUC (0.741) along with superior sensitivity (66.8%), and thus was considered the most effective among all models tested (Fig. 6A and B). To facilitate interpretability of the AdaBoost model, SHAP analyses were conducted at both global and individual levels. The SHAP summary plot identified UAR MoM, TyG MoM, PALB, GGT, and CRP as key contributors to AdaBoost model predictions. High values of these variables were associated with increased PE risk (Fig. 6C). In an individual case, elevated CRP, PALB, and TyG MoM increased predicted risk, whereas low UAR MoM reduced it, consistent with global feature importance (Fig. 6D).

For term PE, the Boruta algorithm identified 19 of the 26 differential features as important, which were subsequently used for model development. Among all models evaluated, LightGBM demonstrated the best overall performance in the testing set for screening purposes. It achieved a sensitivity of 53.8%, an AUC of 0.689, and a balanced accuracy of 68.2%, with minimal evidence of overfitting (Fig. 6E and F). To enhance the interpretability of the LightGBM model, SHAP analyses were performed at both population and individual levels. The SHAP summary plot revealed TyG MoM, PALB, GGT, and CRP as the most influential predictors in the LightGBM model. Elevated levels of TyG MoM and PALB were associated with increased PE risk, while higher GGT and Ca levels demonstrated protective effects (Fig. 6G). In a representative case analysis, increased TyG MoM and PALB values contributed positively to the risk prediction, whereas reduced GGT and elevated Ca levels decreased the predicted risk, aligning with the overall feature importance trends (Fig. 6H).

### Integration of routine laboratory markers and maternal risk profiles for preeclampsia early prediction

Among the cohort, 5901 pregnant women underwent PE risk prediction during pregnancy using the FMF model, which incorporated maternal risk factors as well as MAP, PLGF, and UtA-PI. This prompted an investigation into whether routine laboratory markers could be integrated with the aforementioned parameters to enhance prediction performance through machine learning approaches.

**Figure 7:**
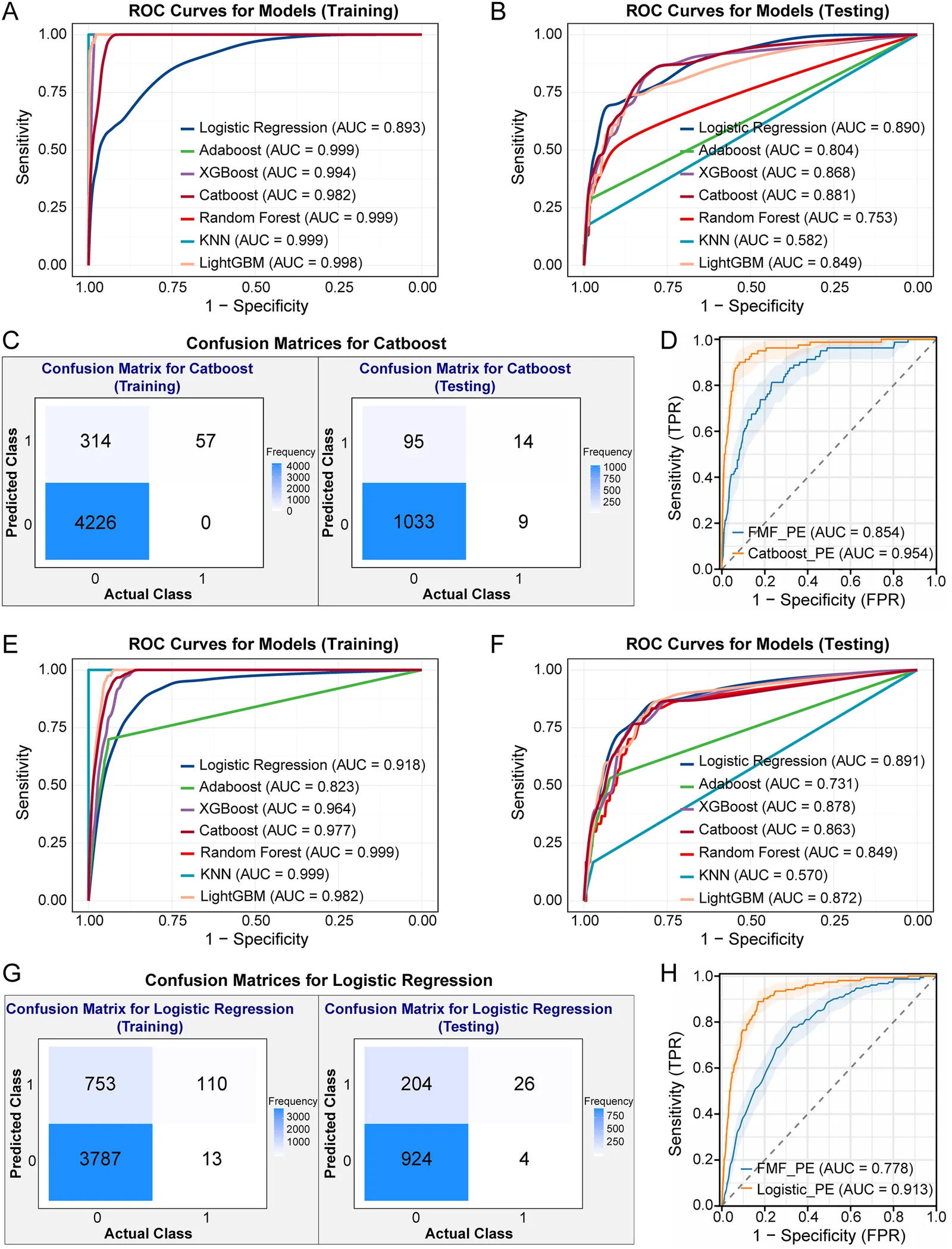
Integration of laboratory markers and maternal risk profiles to predict PE. **(A)** The ROC curves of the preterm PE prediction models in the training set. **(B)** The ROC curves of the preterm PE prediction models in the testing set. **(C)** Confusion matrix analysis of CatBoost model for preterm PE. For the confusion matrix: *X*-axis (actual class): Label “0″ denotes women clinically diagnosed without PE (non-PE cases) and label “1″ denotes women clinically diagnosed with PE (PE cases); *Y*-axis (predicted class): Label “0″ indicates a model prediction of non-PE (low risk cases) and label “1″ indicates a model prediction of PE (high risk cases). **(D)** The ROC curves of CatBoost and FMF model for preterm PE in the entire cohort. **(E)** The ROC curves of the term PE prediction models in the training set. **(F)** The ROC curves of the term PE prediction models in the testing set. **(G)** Confusion matrix analysis of logistic regression model for term PE. **(H)** The ROC curves of logistic regression and FMF model for term PE in the entire cohort. PE, preeclampsia; ROC, receiver operating characteristic; FMF, fetal medicine foundation.

For preterm PE, the highest AUC (0.881) and sensitivity (60.9%) were achieved by the Catboost model in the testing set, making this the most effective among the models evaluated (Fig. 7A–C).

When applied to the entire study population, Catboost's AUC rose to 0.954 (95% CI, 0.914–0.969), a marked improvement over the established FMF model (AUC = 0.854; 95% CI, 0.799–0.857; *p* < 0.001) (Fig. 7D). Moreover, at a prespecified false-positive rate of 14%, the Catboost model detected 92.5% of preterm PE cases, compared with just 65.0% sensitivity for the FMF algorithm. These findings underscore the potential of machine learning-derived risk stratification to substantially enhance early identification of women at highest risk for preterm PE, particularly in settings where the incremental gain in sensitivity could translate into timely prophylactic interventions and improved maternal–fetal outcomes.

For term PE, the logistic regression model demonstrated the highest AUC (0.891) and sensitivity (86.7%) in the testing set, and thus was considered the most effective among all models assessed (Fig. 7E–G). When applied to the entire cohort, the AUC of the logistic regression model increased to 0.913 (95% CI, 0.887–0.931), which greatly exceeded the AUC of the FMF algorithm of 0.778 (95% CI, 0.738–0.810; *p* < 0.001) (Fig. 7H). At a fixed false-positive rate of 14%, the logistic model identified 81.1% of term PE cases, whereas the sensitivity of the FMF model was only 47.7%. These results suggest that a simplified regression-based approach is promising to augment existing FMF screening and thereby significantly improve the early prediction of term PE.

### Early transcriptomic alterations reveal multiple-system dysregulation in preeclampsia

To elucidate the mechanisms underlying early laboratory markers changes in pregnancy, low-coverage whole-genome sequencing was applied to compare cfDNA coverage across TSSs between cases and controls. Reduced promoter coverage inversely reflected higher gene expression in maternal or placental tissues.^9^^,^^10^^,^^15^ This allowed for a noninvasive differential expression analysis, which revealed early transcriptional alterations associated with pregnancies that later developed PE.

**Figure 8:**
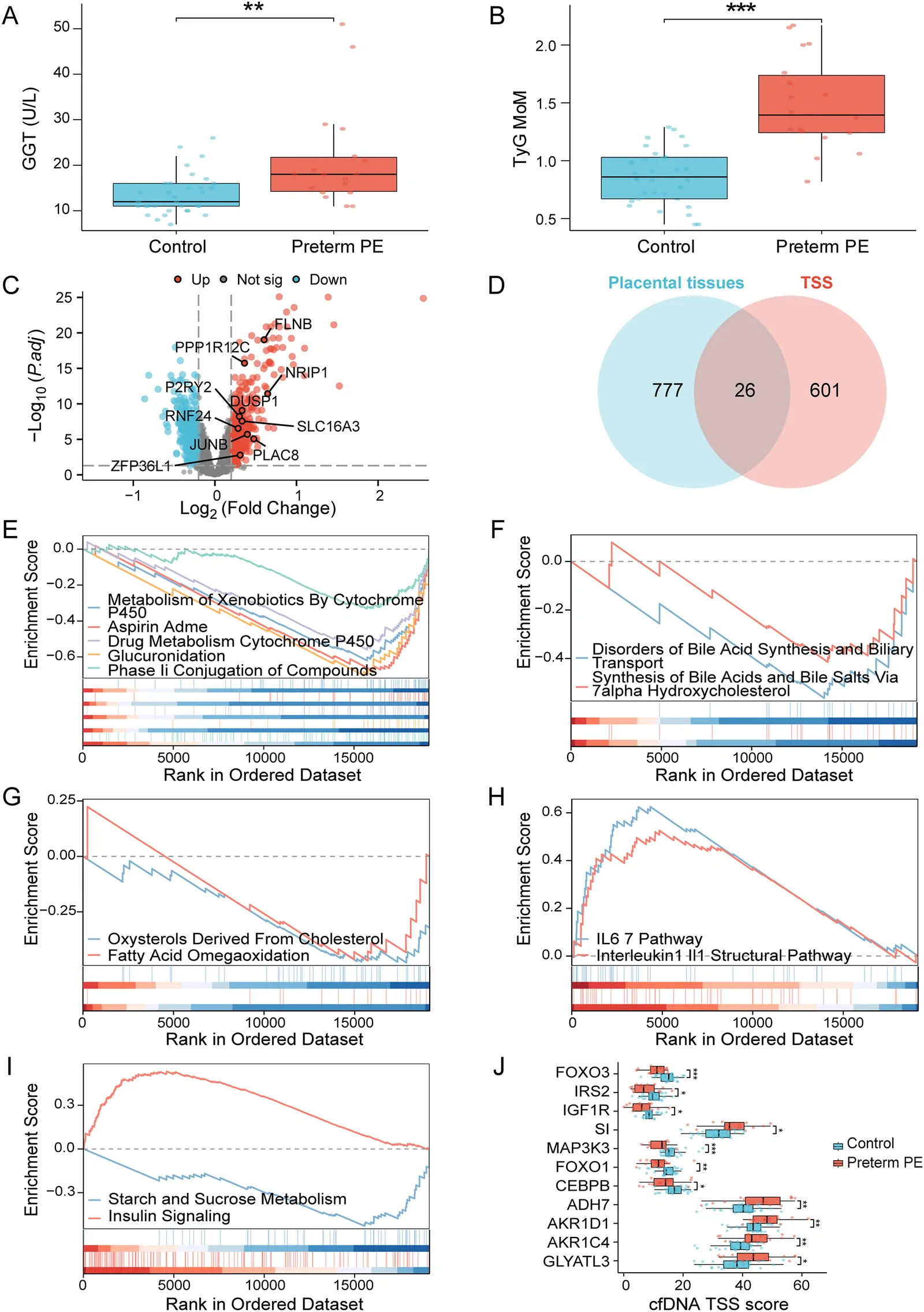
Early transcriptomic alterations reveal multiple-system dysregulation in pregnancies that develop PE. **(A)** Compared with control, women who later developed preterm PE had significantly higher GGT in early pregnancy. **(B)** Compared with control, women who later developed preterm PE had significantly higher TyG MoM in early pregnancy. **(C)** Volcano plot of DEGs (|log2 FC| > 0.2, *P*.adj < 0.05). The red indicates up-regulated genes and the blue indicates down-regulated genes between PE and control groups in the GEO datasets. **(D)** Intersection of GEO-derived placental tissue DEGs and mid-pregnancy cfDNA-derived DEGs. **(E)** GSEA analysis showed significant negative enrichment of pathways related to hepatic function (metabolism of xenobiotics by cytochrome P450, aspirin metabolism, drug metabolism cytochrome P450, glucuronidation, phase II conjugation of compounds) in PE cases. **(F)** GSEA analysis showed significant negative enrichment of pathways related to bile acid synthesis and clearance (disorders of bile acid synthesis and biliary transport, synthesis of bile acids and bile salts via 7 alpha hydroxycholesterol) in PE cases. **(G)** GSEA analysis showed significant negative enrichment of pathways related to lipid oxidation (oxysterols derived from cholesterol, fatty acid omega oxidation) in PE cases. **(H)** GSEA analysis showed significant up-regulation of pro-inflammatory signaling (IL-6 pathway, IL-1 structural pathway) in PE cases. **(I)** GSEA analysis showed that starch and sucrose metabolism pathways were significantly down-regulated, and insulin signaling pathways were significantly up-regulated in PE cases. **(J)** Differential promoter coverage of functionally relevant genes between control and preterm PE cases. PE, preeclampsia; GGT, gamma-glutamyltransferase; TyG MoM, triglyceride-glucose index multiple of the median; DEGs, differentially expressed genes; GSEA, gene set enrichment analysis; cfDNA, cell-free DNA; TSS, transcription start site. ∗*p* < 0.05, ∗∗*p* < 0.01, and ∗∗∗*p* < 0.001.

In total, 51 maternal plasma samples underwent low-depth cfDNA sequencing and TSS profiling, including 18 cases diagnosed with preterm PE—a subtype strongly associated with adverse pregnancy outcomes. Compared with controls, women who later developed preterm PE exhibited significant early pregnancy alterations in GGT and the TyG MoM (Fig. 8A and B). Three datasets were downloaded from GEO, and 803 DEGs were identified in placentas from women with PE using the thresholds of *P*.adj < 0.05 and |log2 fold change| > 0.2 (Fig. 8C and D). In mid-pregnancy cfDNA analysis, 627 DEGs were obtained under the *P*.adj < 0.05 criterion. Intersection analysis revealed 26 overlapping genes, suggesting that TSS signals can reflect early functional changes in the placenta during pregnancy. In particular, expression of the FLNB gene was markedly up-regulated in placental tissue, consistent with prior reports,^16^ whereas it was down-regulated at the TSS level, representing a concordant pattern. Because approximately 10% of cfDNA is derived from the placenta, with the majority originating from maternal leukocytes and the liver, cfDNA analysis may also capture functional alterations in these maternal tissues and organs. Functional enrichment using GSEA revealed coordinated disruptions in key biological processes, including hepatic metabolic dysfunction, lipid processing abnormalities, and systemic inflammatory activation, all of which aligned with routine laboratory markers perturbations observed in early pregnancy in individuals who subsequently developed PE.

Gene sets related to cytochrome P450-mediated metabolism, glucuronidation, aspirin metabolism, drug metabolism via cytochrome P450, and phase II conjugation enzymes were significantly negatively enriched (Fig. 8E). This broad suppression of hepatic enzymatic pathways was interpreted as indicative of reduced maternal capacity to metabolize endogenous waste products and oxidative metabolites. As a result, the accumulation of toxic lipid intermediates, uremic solutes, and pro-inflammatory mediators may occur, imposing additional stress on the maternal vasculature and placenta. The increased cfDNA representation of ADH7 and GLYATL3, reflecting reduced gene expression in contributing tissues, was consistent with clinical observations of elevated liver enzymes (*e.g.*, ALT, GGT, ALP) during early pregnancy in individuals who later developed PE (Fig. 8J).

Impaired detoxification also appeared to affect bile acid synthesis and clearance. Notably, the biliary transport pathway and the synthesis of bile acids and bile salts via the 7α-hydroxycholesterol pathway were negatively enriched, implicating genes such as AKR1C4 and AKR1D1 as playing critical roles in bile acid biosynthesis and cholesterol elimination (Fig. 8F, J). The repression of these pathways may contribute to the dyslipidemic phenotype frequently observed in PE.

Similarly, down-regulation of the oxysterols derived from the cholesterol pathway (NES = −2.22) and the fatty acid ω-oxidation pathway (NES = −1.57) was interpreted as indicative of impaired lipid oxidation and cholesterol derivative metabolism (Fig. 8G). Genes such as ADH7 and AKR1C4 were consistently up-regulated in cfDNA profiles, reflecting reduced expression in contributing tissues and suggesting dysfunctional energy metabolism in both trophoblasts and the maternal liver (Fig. 8J). These metabolic disruptions may lead to lipid accumulation, placental lipotoxicity, and the systemic dyslipidemia characteristic of PE.

In contrast, pro-inflammatory signaling was markedly up-regulated in PE cases. Significant enrichment of the interleukin-6 (IL-6) signaling pathway (NES = +1.95) and the interleukin-1 (IL-1) structural signaling pathway (NES = +2.00) was observed (Fig. 8H). These pathways featured key immune regulators, including CEBPB, FOXO1, and MAP3K3 (Fig. 8J). The activation of these pathways suggested a systemic inflammatory state during early pregnancy, likely promoting endothelial activation, immune cell infiltration, and placental dysfunction.

Metabolic pathway analysis revealed concurrent evidence of glucose handling defects and insulin signaling dysregulation. The starch and sucrose metabolism pathway (NES = −2.51; *p* < 0.001) was significantly down-regulated in PE cases, with key enzymes such as SI involved in glycolysis and carbohydrate interconversion found to be transcriptionally repressed (Fig. 8I and J). These findings suggest reduced glucose utilization and impaired carbohydrate flux, consistent with early insulin resistance. Conversely, the insulin signaling pathway was significantly up-regulated (NES = +2.06; *p* < 0.001), with core enriched genes including IGF1R, IRS2, and FOXO3 mediating canonical insulin signaling (Fig. 8I and J). Although this transcriptional up-regulation may initially suggest enhanced pathway activity, it more likely reflects a compensatory response to impaired insulin sensitivity.

## Discussion

Based on laboratory markers profiles from 12,715 women in early pregnancy, this study systematically identified multiple routine laboratory markers independently linked to the subsequent development of PE, providing fresh insights for early risk evaluation. For the first time, liver function indices (GGT, ALP, CHE), renal marker (UAR MoM), lipid-inflammatory indicators (TyG MoM, CRP), and micronutrient levels (Cu, Ca, Mg, Fe) were shown to be significantly correlated with the risk of subsequent PE development during the early stages of pregnancy. Based on these features, a machine learning model was established and combined with the FMF prediction algorithm, substantially enhancing the model's discriminative performance. Collectively, our findings emphasize that routinely available laboratory markers can serve as a practical, low-cost approach for identifying women at elevated risk of PE, particularly in primary or resource-limited settings.

Regarding demographic characteristics, women who developed PE were slightly older (mean age, 32 *vs*. 31 years) and had a higher median BMI (24.1 *vs*. 21.79 kg/m^2^) than controls, suggesting that advanced maternal age and obesity may jointly contribute to PE pathogenesis through impaired placental vascular remodeling and enhanced systemic inflammatory responses.^17^^,^^18^ Furthermore, higher rates of twin gestation (9.71%) and assisted conception (24.28%) were observed in the PE group, consistent with previous findings.^19^^,^^20^ The combined effects of increased placental mass in multiple pregnancies and hormonal alterations during IVF conception may exacerbate placental ischemia and hypoxia, thereby promoting PE development.^21^, ^22^, ^23^

In this study, women who developed PE exhibited abnormalities in several liver function markers during early pregnancy (< 14 weeks), suggesting that hepatic dysfunction may represent not only a concomitant alteration but also an early indicator or potential contributor to PE pathogenesis. Mild elevations in ALT and GGT were interpreted as evidence of subclinical hepatocellular injury or cholestasis, which may aggravate placental oxidative stress through the systemic release of mitochondrial reactive oxygen species reaching the placenta via the circulation.^24^, ^25^, ^26^, ^27^ In addition, elevated bile acid levels may disrupt placental development through multiple mechanisms: by activating the farnesoid X receptor (FXR) and its downstream effector, the small heterodimer partner (SHP), within the placenta, leading to reduced VEGF and PLGF expression and impaired angiogenesis;^28^, ^29^, ^30^ and by promoting mitochondrial membrane permeability via hydrophobic bile acids such as deoxycholic acid, thereby triggering chromogranin C release, apoptotic signaling, and trophoblast cell death.^31^^,^^32^ Concurrently, elevated UAR MoM (OR = 10.16) emerged as another significant indicator of PE risk, consistent with recent Mendelian randomization evidence linking early pregnancy hyperuricemia to a 1.21-fold increased risk of PE.^33^

Disordered lipid metabolism has been increasingly recognized as a key metabolic feature of PE. The TyG MoM (OR = 4.29) was identified as an independent risk factor, suggesting the contribution of insulin resistance to its pathogenesis. Hyperinsulinemia may induce vasoconstriction and impair placental perfusion by suppressing nitric oxide synthesis and enhancing endothelin-1 release, thereby exacerbating endothelial dysfunction.^34^^,^^35^ In parallel, elevated TyG levels may aggravate oxidative stress and systemic inflammation via NADPH oxidase activation, leading to excessive reactive oxygen species generation and increased secretion of pro-inflammatory cytokines such as IL-6 and tumor necrosis factor-α (TNF-α) from adipose tissue.^36^^,^^37^ Functional impairment of HDL reduces its anti-inflammatory and cholesterol efflux capacities, whereas oxidative modification of LDL and VLDL generates oxidized LDL (ox-LDL), which activates macrophages and promotes foam-cell formation, leading to atherosclerosis-like remodeling of placental vessels.^38^, ^39^, ^40^ Collectively, these findings indicate that insulin resistance and lipid disorders act synergistically accelerate vascular injury in PE.

Electrolyte disturbances were also observed, characterized by increased Na, K, Ca, and Cu levels and decreased Mg and Fe levels, reflecting oxidative stress and impaired placental perfusion. Interestingly, Mg concentrations below a defined threshold showed an inverse association with PE risk, suggesting that higher Mg levels may confer vascular protection by limiting calcium entry and alleviating smooth muscle tension through activation of transient receptor potential melastatin 7 (TRPM7) channels.^41^^,^^42^ Finally, CRP exhibited a dose-dependent biphasic association with PE: below 8.09 mg/L, each 1 mg/L increase corresponded to a higher PE risk (OR = 1.18), indicating its dual role as a marker of vascular inflammation and a mediator of endothelial dysfunction. Collectively, this study demonstrates that hepatic dysfunction, insulin resistance, lipid dysregulation, electrolyte imbalance, and inflammation converge in the pathogenesis of PE. These findings reinforce the notion that PE is not merely a placental disorder but a systemic condition arising from early metabolic, immune, and vascular dysfunctions.^43^^,^^44^ Consequently, redefining intervention windows and preventive strategies from a systemic perspective centered on these early alterations may help improve maternal outcomes.

In multicenter studies conducted among Asian populations (*e.g.*, in China), the FMF competing risk model yields a detection rate of about 60%–82% for predicting preterm PE,^45^^,^^46^ which is marginally lower than that reported in Caucasian and African cohorts. This disparity was also observed in the present study. Previous research has reported systematic differences in the regression patterns of MoM values for biomarkers such as MAP and UtA-PI across ethnic groups. Specifically, compared with the FMF reference population, East Asian women generally exhibit steeper declines in MAP MoM and flatter trends in UtA-PI MoM,^47^^,^^48^ and these non-compensatory differences may partly explain the reduced predictive efficiency of the FMF model in Asian populations. Furthermore, the FMF model primarily incorporates biophysical, biochemical, and ultrasonographic parameters in early pregnancy, while largely neglecting maternal factors such as inflammation, oxidative stress, and metabolic dysfunction—conditions more common in older, overweight, or IVF conception, particularly among Asian women. In this study, integrating routine laboratory markers, including liver and renal function tests, electrolytes, and lipid profiles, broadened the model's dimensional scope without increasing cost, providing a more practical and cost-effective screening strategy for resource-limited settings. For preterm PE, integrating conventional laboratory markers with the FMF model (MAP, PLGF, and UtA-PI) substantially enhanced predictive performance. The combined model achieved an AUC of 0.954 (95% CI, 0.914–0.969), significantly higher than the FMF model alone (AUC = 0.854, *p* < 0.001), with the detection rate rising to 92.5% from 65.0% at a 14% false-positive rate. For term PE, the logistic regression model yielded an AUC of 0.913 (95% CI, 0.887–0.931), outperforming the FMF model (AUC = 0.778, *p* < 0.001), and sensitivity increased to 81.1% from 47.7% at the same false-positive rate. SHAP analysis further revealed that inflammatory and metabolic markers contributed most strongly to model output, addressing the FMF model's limited responsiveness to such abnormalities and offering a plausible explanation for the superior predictive capacity of the integrated approach.

To implement the combined model in low-resource settings, we propose a minimal test set that includes key laboratory markers (GGT, ALP, UAR MoM, TyG MoM, CRP, Ca, and Mg) alongside FMF model parameters (maternal risk factors, MAP, PLGF, and UtA-PI). This streamlined panel balances strong predictive power with cost-effectiveness, making it feasible in settings with limited infrastructure. Clinically, the model can guide early management by stratifying risk. High-risk women identified through this model can be prioritized for closer monitoring, early initiation of low-dose aspirin (if appropriate), and more frequent assessment of blood pressure and fetal growth. Identifying specific metabolic issues, such as an elevated TyG index or low Mg, may prompt targeted interventions like dietary counseling or nutrient supplementation. A phased screening approach allows adaptive resource allocation: primary care settings can perform initial assessments, referring high-risk cases to higher-level facilities for confirmatory tests. This ensures efficient use of resources and timely interventions. The model can also be deployed via mobile platforms, enabling accessible, low-cost risk assessment without specialized equipment.

cfDNA TSS profiling in mid-pregnancy revealed extensive metabolic and inflammatory disturbances preceding the clinical onset of preterm PE. Functional enrichment analyses demonstrated coordinated down-regulation of hepatic detoxification pathways, including cytochrome P450 metabolism, glucuronidation, bile acid synthesis, and aspirin metabolism, suggesting reduced maternal capacity for endogenous toxin clearance and aligning with the preventive efficacy of early aspirin administration. Simultaneous up-regulation of IL-1 and IL-6 signaling suggested a systemic inflammatory milieu that may contribute to endothelial dysfunction and placental injury. Suppression of glycolytic and glucose handling genes, along with paradoxical activation of insulin signaling components (*e.g.*, IGF1R, IRS2), implied early insulin resistance with compensatory transcriptional responses. These transcriptomic signatures were consistent with routine laboratory markers abnormalities observed in individuals who later developed PE, including elevated GGT, increased TyG index, and subclinical inflammation. Collectively, cfDNA TSS profiling enabled early detection of multisystem dysregulation, offering mechanistic insights into PE pathogenesis and a noninvasive means for early risk stratification and intervention.

Although retrospective, this multicenter study with a large cohort provides meaningful insights. Nonetheless, several limitations should be noted. The study cohort is drawn exclusively from Chinese pregnant women, and laboratory marker distributions and reference intervals may vary substantially across populations due to ethnic, genetic, dietary, and environmental differences. This variability underscores the challenges of transferring the model to different populations. The inconsistent performance of traditional tools such as the FMF algorithm in non-European (*e.g.*, Asian) populations highlights the necessity for population-tailored or globally adaptable frameworks to achieve equitable, context-aware risk prediction in diverse clinical settings. Consequently, the risk estimates and biomarker thresholds identified in our model may not directly apply to non-Chinese populations without further calibration. External validation using independent prospective cohorts, particularly from diverse communities and primary care settings, is essential to confirm the model's generalizability and real-world applicability. Additionally, all laboratory markers were assessed at a single time point; future work should determine whether longitudinal trajectories, particularly in later trimesters, enhance predictive accuracy. Despite these limitations, the pathophysiological pathways emphasized by our model—liver dysfunction, dyslipidemia, systemic inflammation, and insulin resistance—are widely recognized in the pathogenesis of PE. Therefore, while specific thresholds may need adjustment, the overall approach of integrating routine laboratory markers with clinical parameters is likely to remain relevant across different populations.

In this study of 12,715 pregnancies, early metabolic, inflammatory, and laboratory markers, including liver enzymes, cholesterol, CRP, TyG index, and UAR, were significantly linked to PE risk, with several showing non-linear threshold effects. Machine learning models constructed from these routine laboratory indicators outperformed the traditional FMF model in predicting both preterm and term PE. SHAP analysis further confirmed the interpretability of the model and the relevance of key features. Overall, integrating routine laboratory parameters into machine learning frameworks may improve early PE screening, enable individualized interventions, and benefit maternal–fetal outcomes. Future studies should focus on validating this approach across diverse populations and evaluating its feasibility in clinical implementation.

## CRediT authorship contribution statement

**Yi Zhu:** Writing – review & editing, Writing – original draft, Visualization, Validation, Software, Resources, Methodology, Funding acquisition, Formal analysis, Data curation, Conceptualization. **Yanqiu Zhang:** Writing – review & editing, Validation, Methodology, Investigation, Data curation. **Chao Huang:** Writing – review & editing, Visualization, Resources, Formal analysis, Data curation. **Sheng Zhang:** Validation, Methodology, Formal analysis. **Jun Cao:** Writing – review & editing, Visualization, Validation, Methodology. **Nicole Miranda:** Writing – review & editing, Visualization, Methodology, Investigation. **Sarina Zhao:** Writing – review & editing, Methodology, Investigation. **Yan Peng:** Writing – review & editing, Investigation, Data curation. **Chao Yu:** Writing – review & editing, Methodology, Formal analysis. **Bin Feng:** Software, Resources, Methodology. **Jieyu Jin:** Writing – review & editing, Data curation. **Qingqin Tang:** Writing – review & editing, Resources, Formal analysis. **Jiaming Fan:** Writing – review & editing, Visualization, Validation, Investigation, Data curation. **Longwei Qiao:** Writing – review & editing, Writing – original draft, Resources, Project administration, Investigation, Funding acquisition. **Yuting Liang:** Writing – review & editing, Writing – original draft, Supervision, Project administration, Investigation, Funding acquisition, Conceptualization.

## Ethics declaration

This study was reviewed and approved by the Reproductive Medicine Ethics Committee of Suzhou Municipal Hospital (ID: K-2025-200-K01). All procedures of this retrospective cohort study, including data collection, de-identification, and outcome analysis, were conducted in compliance with the Helsinki Declaration. Written informed consent from the (patients/participants or patients/participants legal guardian/next of kin) was not required to participate in this study in accordance with the national legislation and the institutional requirements.

## Funding

This study was supported by the Science Foundation of Jiangsu Province (China) (No. BK20240371), the Suzhou Health Talent Program (Jiangsu, China) (No. GSWS2024046), the 10.13039/501100012166National Key R&D Program of China (No. 2023YFC2705600, 2023YFC2705602), the 10.13039/501100001809National Natural Science Foundation of China (No. 82001576), the Postdoctoral Fellowship Program of CPSF (No. GZC20251571), the Jiangsu Funding Program for Excellent Postdoctoral Talent (China) (No. 2025ZB269), Suzhou Science and Technology Support Program (China) (No. SKY2023001, SYW2025137), the 10.13039/501100013058Primary Research and Development Plan of Jiangsu Province, China (No. BE2022736), and the Jiangsu Province College Students' Innovation and Entrepreneurship Training Program Project (China) (No. 202410285273Y).

## Conflict of interests

The authors declared no conflict of interests.
