# Supplementary material for: First-trimester preeclampsia prediction model via integrative machine learning of maternal risk profiles and laboratory markers
Source: Genes Dis. 2026 Feb 9;13(6):102075. doi: 10.1016/j.gendis.2026.102075 (PMC13499121; doi:10.1016/j.gendis.2026.102075)
Supplement: Supplementary file 1 [file mmc1.docx]

**Table S1. Baseline characteristics of pregnant women.**

| Variables | Total (n = 12715)  M (Q₁, Q₃)/n(%) | Control (n = 12159)  M (Q₁, Q₃)/n(%) | PE (n = 556)  M (Q₁, Q₃)/n(%) | *P* |
| --- | --- | --- | --- | --- |
|  |  |  |  |  |
| Gestational age at blood sampling (weeks) | 12.43 (11.81, 13.10) | 12.43 (11.86, 13.10) | 12.29 (11.71, 13.00) | 0.283 |
| Age (years) | 31.00 (28.00, 34.00) | 31.00 (28.00, 34.00) | 32.00 (29.00, 35.00) | <0.001 |
| BMI (kg/m2) | 21.87 (20.21, 23.88) | 21.79 (20.20, 23.73) | 24.10 (21.59, 26.67) | <0.001 |
| Plurality |  |  |  | <0.001 |
| Singleton | 12517 (98.44) | 12015 (98.82) | 502 (90.29) |  |
| Twin | 198 (1.56) | 144 (1.18) | 54 (9.71) |  |
| Gravidity | 2.00 (1.00, 2.00) | 2.00 (1.00, 2.00) | 2.00 (1.00, 2.00) | 0.281 |
| Parity | 0.00 (0.00, 1.00) | 0.00 (0.00, 1.00) | 0.00 (0.00, 1.00) | 0.004 |
| IVF conception |  |  |  | <0.001 |
| No | 11558 (90.90) | 11137 (91.59) | 421 (75.72) |  |
| Yes | 1157 (9.10) | 1022 (8.41) | 135 (24.28) |  |
| TBA (μmol/L) | 1.20 (0.80, 1.90) | 1.20 (0.80, 1.90) | 1.30 (0.80, 2.05) | <0.001 |
| A/G | 1.50 (1.40, 1.70) | 1.50 (1.40, 1.70) | 1.50 (1.40, 1.70) | 0.013 |
| PALB (mg/L) | 226.40 (207.60, 246.60) | 225.80 (207.10, 246.00) | 239.45 (220.20, 262.20) | <0.001 |
| TP (g/L) | 73.50 (70.90, 76.00) | 73.50 (70.90, 76.00) | 73.60 (71.10, 76.30) | 0.357 |
| ALB (g/L) | 44.60 (42.90, 46.40) | 44.70 (42.90, 46.40) | 44.50 (42.70, 46.20) | 0.103 |
| GLB (g/L) | 28.90 (26.70, 31.00) | 28.90 (26.70, 31.00) | 29.15 (27.17, 31.20) | 0.018 |
| AST (U/L) | 18.00 (15.00, 21.00) | 18.00 (15.00, 21.00) | 18.00 (15.00, 22.00) | 0.085 |
| ALT (U/L) | 14.00 (11.00, 21.00) | 14.00 (10.00, 21.00) | 17.00 (12.00, 26.00) | <0.001 |
| ALP (U/L) | 51.00 (45.00, 59.00) | 51.00 (44.00, 59.00) | 55.00 (47.00, 63.00) | <0.001 |
| GGT (U/L) | 13.00 (11.00, 18.00) | 13.00 (10.00, 18.00) | 18.00 (12.00, 27.00) | <0.001 |
| LDH (U/L) | 164.00 (149.00, 180.00) | 164.00 (149.00, 180.00) | 166.00 (152.00, 186.00) | <0.001 |
| CHE (U/L) | 294.90 (262.00, 331.00) | 293.00 (261.00, 329.00) | 321.00 (284.75, 366.00) | <0.001 |
| TBIL (μmol/L) | 8.60 (7.20, 10.60) | 8.60 (7.20, 10.60) | 8.40 (7.00, 10.22) | 0.009 |
| DBIL (μmol/L) | 1.90 (1.50, 2.40) | 1.90 (1.50, 2.40) | 1.90 (1.40, 2.40) | 0.038 |
| IBIL (μmol/L) | 6.70 (5.50, 8.30) | 6.70 (5.50, 8.30) | 6.50 (5.30, 8.00) | 0.013 |
| HBDH (U/L) | 132.00 (119.00, 146.00) | 132.00 (119.00, 146.00) | 133.00 (120.00, 146.00) | 0.398 |
| CK (U/L) | 41.00 (33.00, 51.00) | 41.00 (33.00, 50.50) | 40.00 (33.00, 52.00) | 0.512 |
| FBG (mmol/L) | 4.57 (4.31, 4.82) | 4.57 (4.31, 4.81) | 4.66 (4.39, 5.01) | <0.001 |
| UA (μmol/L) | 212.30 (184.10, 242.70) | 211.40 (183.60, 240.90) | 243.00 (206.73, 278.18) | <0.001 |
| Urea (mmol/L) | 2.85 (2.43, 3.34) | 2.85 (2.43, 3.34) | 2.85 (2.41, 3.36) | 0.962 |
| Cr (μmol/L) | 42.00 (38.10, 45.80) | 42.00 (38.10, 45.80) | 42.10 (38.08, 46.30) | 0.285 |
| UAR MoM | 1.00 (0.87, 1.16) | 1.00 (0.86, 1.15) | 1.15 (1.00, 1.31) | <0.001 |
| TG (mmol/L) | 1.29 (1.02, 1.65) | 1.28 (1.02, 1.63) | 1.58 (1.18, 2.06) | <0.001 |
| TyG MoM | 1.01 (0.78, 1.25) | 1.00 (0.78, 1.24) | 1.22 (0.97, 1.48) | <0.001 |
| TC (mmol/L) | 4.63 (4.17, 5.12) | 4.62 (4.16, 5.12) | 4.77 (4.23, 5.25) | <0.001 |
| HDL-C (mmol/L) | 1.78 (1.56, 2.01) | 1.78 (1.56, 2.01) | 1.69 (1.47, 1.95) | <0.001 |
| LDL-C (mmol/L) | 2.40 (2.03, 2.82) | 2.40 (2.03, 2.81) | 2.54 (2.15, 3.03) | <0.001 |
| VLDL-C (mmol/L) | 0.42 (0.32, 0.53) | 0.42 (0.32, 0.53) | 0.47 (0.35, 0.58) | <0.001 |
| CRP (mg/L) | 2.00 (1.00, 3.94) | 1.97 (0.99, 3.88) | 3.06 (1.40, 6.15) | <0.001 |
| CO_2_ (mmol/L) | 22.40 (20.60, 24.40) | 22.50 (20.60, 24.40) | 22.10 (20.25, 24.20) | 0.005 |
| Na (mmol/L) | 139.10 (137.60, 140.50) | 139.10 (137.60, 140.40) | 139.40 (137.90, 140.60) | 0.014 |
| K (mmol/L) | 4.01 (3.85, 4.19) | 4.01 (3.85, 4.19) | 4.03 (3.88, 4.22) | 0.045 |
| Cl (mmol/L) | 103.80 (102.10, 105.00) | 103.90 (102.10, 105.00) | 103.55 (102.00, 105.00) | 0.174 |
| Ca (mmol/L) | 2.28 (2.23, 2.35) | 2.28 (2.23, 2.35) | 2.30 (2.24, 2.36) | 0.002 |
| P (mmol/L) | 1.26 (1.17, 1.36) | 1.27 (1.17, 1.36) | 1.25 (1.15, 1.36) | 0.075 |
| Mg (mmol/L) | 0.86 (0.82, 0.90) | 0.86 (0.82, 0.90) | 0.84 (0.80, 0.89) | <0.001 |
| Cu (μmol/L) | 27.72 (24.53, 31.10) | 27.69 (24.52, 31.05) | 28.62 (24.92, 32.86) | <0.001 |
| Fe (μmol/L) | 21.20 (17.20, 25.00) | 21.20 (17.30, 25.00) | 20.50 (15.70, 24.55) | 0.001 |
| Zn (μmol/L) | 14.70 (12.80, 16.40) | 14.70 (12.80, 16.40) | 14.50 (12.80, 16.20) | 0.518 |

M (Q₁, Q₃), median (first quartile, third quartile); n(%), numbers (percentages); PE, preeclampsia; BMI, body mass index; IVF, in vitro fertilization; TBA, total bile acids; A/G, albumin-to-globulin ratio; PALB, prealbumin; TP, total protein; ALB, albumin; GLB, globulin; AST, aspartate transaminase; ALT, alanine aminotransferase; ALP, alkaline phosphatase; GGT, gamma-glutamyltransferase; LDH, lactate dehydrogenase; CHE, cholinesterase; TBIL, total bilirubin; DBIL, direct bilirubin; IBIL, indirect bilirubin; HBDH, hydroxybutyrate dehydrogenase; CK, creatine kinase; FBG, fasting blood glucose; UA, uric acid; Cr, creatinine; UAR MoM, uric acid-albumin ratio multiple of the median; TG, triglycerides; TyG MoM, triglyceride-glucose index multiple of the median; TC, total cholesterol; HDL-C, high-density lipoprotein cholesterol; LDL-C, low-density lipoprotein cholesterol; VLDL-C, very low-density lipoprotein cholesterol; CRP, c-reactive protein; CO_2_, total carbon dioxide; Na, sodium; K, potassium; Cl, chloride; Ca, calcium; P, phosphorus; Mg, magnesium; Cu, copper; Fe, iron; Zn, zinc.

**Table S2. Association between** **laboratory markers in early pregnancy and the risk of PE.**

| Variables | Model1 | |  | Model2 | |  | Model3 | |
| --- | --- | --- | --- | --- | --- | --- | --- | --- |
|  | OR (95%CI) | *P* |  | OR (95%CI) | *P* |  | OR (95%CI) | *P* |
| PALB | 1.01 (1.01 ~ 1.02) | <0.001 |  | 1.01 (1.01 ~ 1.02) | <0.001 |  | 1.01 (1.01 ~ 1.02) | <0.001 |
| PALB (Quartile) |  |  |  |  |  |  |  |  |
| 1 | 1.00 (Reference) |  |  | 1.00 (Reference) |  |  | 1.00 (Reference) |  |
| 2 | 1.18 (0.88 ~ 1.58) | 0.275 |  | 1.19 (0.88 ~ 1.60) | 0.254 |  | 1.17 (0.87 ~ 1.57) | 0.302 |
| 3 | 1.63 (1.24 ~ 2.15) | <0.001 |  | 1.60 (1.21 ~ 2.12) | <0.001 |  | 1.53 (1.16 ~ 2.03) | 0.003 |
| 4 | 2.96 (2.30 ~ 3.82) | <0.001 |  | 2.81 (2.17 ~ 3.63) | <0.001 |  | 2.62 (2.02 ~ 3.40) | <0.001 |
| *P* for trend |  | <0.001 |  |  | <0.001 |  |  | <0.001 |
| GLB | 1.03 (1.01 ~ 1.05) | 0.035 |  | 1.02 (1.00 ~ 1.05) | 0.072 |  | 1.03 (1.01 ~ 1.05) | 0.046 |
| GLB (Quartile) |  |  |  |  |  |  |  |  |
| 1 | 1.00 (Reference) |  |  | 1.00 (Reference) |  |  | 1.00 (Reference) |  |
| 2 | 1.30 (1.01 ~ 1.68) | 0.040 |  | 1.30 (1.01 ~ 1.68) | 0.044 |  | 1.31 (1.01 ~ 1.69) | 0.039 |
| 3 | 1.28 (0.99 ~ 1.65) | 0.059 |  | 1.21 (0.94 ~ 1.57) | 0.144 |  | 1.21 (0.93 ~ 1.56) | 0.152 |
| 4 | 1.42 (1.11 ~ 1.82) | 0.005 |  | 1.37 (1.06 ~ 1.75) | 0.014 |  | 1.40 (1.09 ~ 1.80) | 0.009 |
| *P* for trend |  | 0.010 |  |  | 0.030 |  |  | 0.020 |
| ALT | 1.01 (1.01 ~ 1.01) | <0.001 |  | 1.01 (1.01 ~ 1.01) | <0.001 |  | 1.01 (1.01 ~ 1.01) | 0.008 |
| ALT (Quartile) |  |  |  |  |  |  |  |  |
| 1 | 1.00 (Reference) |  |  | 1.00 (Reference) |  |  | 1.00 (Reference) |  |
| 2 | 1.17 (0.89 ~ 1.55) | 0.263 |  | 1.18 (0.89 ~ 1.56) | 0.261 |  | 1.13 (0.85 ~ 1.50) | 0.393 |
| 3 | 1.29 (1.00 ~ 1.67) | 0.052 |  | 1.23 (0.94 ~ 1.59) | 0.125 |  | 1.19 (0.91 ~ 1.54) | 0.203 |
| 4 | 2.02 (1.58 ~ 2.57) | <0.001 |  | 1.90 (1.49 ~ 2.43) | <0.001 |  | 1.81 (1.41 ~ 2.32) | <0.001 |
| *P* for trend |  | <0.001 |  |  | <0.001 |  |  | <0.001 |
| ALP | 1.02 (1.01 ~ 1.03) | <0.001 |  | 1.02 (1.01 ~ 1.03) | <0.001 |  | 1.02 (1.02 ~ 1.03) | <0.001 |
| ALP (Quartile) |  |  |  |  |  |  |  |  |
| 1 | 1.00 (Reference) |  |  | 1.00 (Reference) |  |  | 1.00 (Reference) |  |
| 2 | 1.00 (0.76 ~ 1.33) | 0.974 |  | 1.00 (0.76 ~ 1.34) | 0.973 |  | 1.03 (0.77 ~ 1.37) | 0.863 |
| 3 | 1.26 (0.97 ~ 1.62) | 0.083 |  | 1.26 (0.97 ~ 1.64) | 0.079 |  | 1.34 (1.03 ~ 1.74) | 0.028 |
| 4 | 1.89 (1.49 ~ 2.40) | <0.001 |  | 1.89 (1.49 ~ 2.40) | <0.001 |  | 2.02 (1.59 ~ 2.58) | <0.001 |
| *P* for trend |  | <0.001 |  |  | <0.001 |  |  | <0.001 |
| GGT | 1.03 (1.03 ~ 1.04) | <0.001 |  | 1.03 (1.03 ~ 1.04) | <0.001 |  | 1.03 (1.02 ~ 1.03) | <0.001 |
| GGT (Quartile) |  |  |  |  |  |  |  |  |
| 1 | 1.00 (Reference) |  |  | 1.00 (Reference) |  |  | 1.00 (Reference) |  |
| 2 | 0.91 (0.66 ~ 1.26) | 0.577 |  | 0.91 (0.66 ~ 1.27) | 0.586 |  | 0.89 (0.64 ~ 1.25) | 0.511 |
| 3 | 1.13 (0.86 ~ 1.50) | 0.374 |  | 1.13 (0.85 ~ 1.49) | 0.394 |  | 1.10 (0.83 ~ 1.45) | 0.524 |
| 4 | 3.09 (2.43 ~ 3.94) | <0.001 |  | 3.08 (2.41 ~ 3.94) | <0.001 |  | 2.86 (2.23 ~ 3.66) | <0.001 |
| *P* for trend |  | <0.001 |  |  | <0.001 |  |  | <0.001 |
| LDH | 1.01 (1.01 ~ 1.01) | <0.001 |  | 1.01 (1.01 ~ 1.01) | <0.001 |  | 1.01 (1.01 ~ 1.01) | 0.002 |
| LDH (Quartile) |  |  |  |  |  |  |  |  |
| 1 | 1.00 (Reference) |  |  | 1.00 (Reference) |  |  | 1.00 (Reference) |  |
| 2 | 1.18 (0.92 ~ 1.52) | 0.190 |  | 1.16 (0.90 ~ 1.49) | 0.256 |  | 1.15 (0.89 ~ 1.49) | 0.280 |
| 3 | 1.05 (0.81 ~ 1.36) | 0.731 |  | 0.98 (0.75 ~ 1.27) | 0.857 |  | 0.95 (0.73 ~ 1.24) | 0.733 |
| 4 | 1.54 (1.22 ~ 1.96) | <0.001 |  | 1.38 (1.08 ~ 1.76) | 0.009 |  | 1.32 (1.04 ~ 1.69) | 0.024 |
| *P* for trend |  | <0.001 |  |  | 0.018 |  |  | 0.046 |
| CHE | 1.01 (1.01 ~ 1.01) | <0.001 |  | 1.01 (1.01 ~ 1.01) | <0.001 |  | 1.01 (1.01 ~ 1.01) | <0.001 |
| CHE (Quartile) |  |  |  |  |  |  |  |  |
| 1 | 1.00 (Reference) |  |  | 1.00 (Reference) |  |  | 1.00 (Reference) |  |
| 2 | 1.27 (0.94 ~ 1.72) | 0.120 |  | 1.26 (0.93 ~ 1.71) | 0.136 |  | 1.26 (0.93 ~ 1.71) | 0.142 |
| 3 | 1.74 (1.31 ~ 2.32) | <0.001 |  | 1.70 (1.27 ~ 2.27) | <0.001 |  | 1.69 (1.26 ~ 2.26) | <0.001 |
| 4 | 3.46 (2.66 ~ 4.49) | <0.001 |  | 3.27 (2.51 ~ 4.26) | <0.001 |  | 3.22 (2.46 ~ 4.19) | <0.001 |
| *P* for trend |  | <0.001 |  |  | <0.001 |  |  | <0.001 |
| DBIL | 0.89 (0.80 ~ 0.99) | 0.032 |  | 0.90 (0.80 ~ 0.99) | 0.046 |  | 0.89 (0.80 ~ 0.99) | 0.043 |
| DBIL (Quartile) |  |  |  |  |  |  |  |  |
| 1 | 1.00 (Reference) |  |  | 1.00 (Reference) |  |  | 1.00 (Reference) |  |
| 2 | 0.72 (0.57 ~ 0.92) | 0.009 |  | 0.75 (0.58 ~ 0.96) | 0.020 |  | 0.74 (0.58 ~ 0.95) | 0.017 |
| 3 | 0.82 (0.65 ~ 1.05) | 0.114 |  | 0.84 (0.66 ~ 1.08) | 0.173 |  | 0.84 (0.66 ~ 1.07) | 0.155 |
| 4 | 0.78 (0.62 ~ 0.98) | 0.035 |  | 0.80 (0.63 ~ 1.01) | 0.061 |  | 0.80 (0.63 ~ 1.01) | 0.057 |
| *P* for trend |  | 0.147 |  |  | 0.197 |  |  | 0.192 |
| UAR-MoM | 11.37 (8.29 ~ 15.61) | <0.001 |  | 10.74 (7.77 ~ 14.84) | <0.001 |  | 10.16 (7.33 ~ 14.09) | <0.001 |
| UAR MoM (Quartile) |  |  |  |  |  |  |  |  |
| 1 | 1.00 (Reference) |  |  | 1.00 (Reference) |  |  | 1.00 (Reference) |  |
| 2 | 1.24 (0.89 ~ 1.74) | 0.210 |  | 1.23 (0.87 ~ 1.72) | 0.235 |  | 1.20 (0.86 ~ 1.69) | 0.286 |
| 3 | 2.13 (1.58 ~ 2.87) | <0.001 |  | 2.07 (1.53 ~ 2.80) | <0.001 |  | 2.03 (1.50 ~ 2.75) | <0.001 |
| 4 | 4.44 (3.37 ~ 5.86) | <0.001 |  | 4.22 (3.19 ~ 5.58) | <0.001 |  | 4.04 (3.06 ~ 5.35) | <0.001 |
| *P* for trend |  | <0.001 |  |  | <0.001 |  |  | <0.001 |
| TyG MoM | 5.05 (4.04 ~ 6.32) | <0.001 |  | 4.37 (3.47 ~ 5.51) | <0.001 |  | 4.29 (3.40 ~ 5.42) | <0.001 |
| TyG MoM (Quartile) |  |  |  |  |  |  |  |  |
| 1 | 1.00 (Reference) |  |  | 1.00 (Reference) |  |  | 1.00 (Reference) |  |
| 2 | 0.88 (0.64 ~ 1.21) | 0.436 |  | 0.85 (0.62 ~ 1.17) | 0.319 |  | 0.86 (0.62 ~ 1.19) | 0.358 |
| 3 | 1.76 (1.33 ~ 2.31) | <0.001 |  | 1.65 (1.25 ~ 2.18) | <0.001 |  | 1.68 (1.27 ~ 2.22) | <0.001 |
| 4 | 3.21 (2.49 ~ 4.14) | <0.001 |  | 2.75 (2.12 ~ 3.56) | <0.001 |  | 2.73 (2.10 ~ 3.55) | <0.001 |
| *P* for trend |  | <0.001 |  |  | <0.001 |  |  | <0.001 |
| TC | 1.24 (1.12 ~ 1.38) | <0.001 |  | 1.19 (1.06 ~ 1.32) | 0.002 |  | 1.17 (1.05 ~ 1.30) | 0.005 |
| TC (Quartile) |  |  |  |  |  |  |  |  |
| 1 | 1.00 (Reference) |  |  | 1.00 (Reference) |  |  | 1.00 (Reference) |  |
| 2 | 1.05 (0.81 ~ 1.36) | 0.712 |  | 1.05 (0.81 ~ 1.36) | 0.722 |  | 1.05 (0.81 ~ 1.36) | 0.732 |
| 3 | 1.17 (0.91 ~ 1.51) | 0.207 |  | 1.15 (0.90 ~ 1.49) | 0.268 |  | 1.13 (0.88 ~ 1.46) | 0.348 |
| 4 | 1.45 (1.14 ~ 1.84) | 0.002 |  | 1.35 (1.06 ~ 1.71) | 0.016 |  | 1.32 (1.04 ~ 1.69) | 0.025 |
| *P* for trend |  | <0.001 |  |  | 0.009 |  |  | 0.016 |
| HDL-C | 0.46 (0.36 ~ 0.60) | <0.001 |  | 0.46 (0.36 ~ 0.60) | <0.001 |  | 0.48 (0.37 ~ 0.63) | <0.001 |
| HDL-C (Quartile) |  |  |  |  |  |  |  |  |
| 1 | 1.00 (Reference) |  |  | 1.00 (Reference) |  |  | 1.00 (Reference) |  |
| 2 | 0.76 (0.61 ~ 0.95) | 0.014 |  | 0.78 (0.62 ~ 0.97) | 0.029 |  | 0.77 (0.62 ~ 0.97) | 0.027 |
| 3 | 0.57 (0.45 ~ 0.73) | <0.001 |  | 0.58 (0.46 ~ 0.74) | <0.001 |  | 0.59 (0.46 ~ 0.75) | <0.001 |
| 4 | 0.57 (0.45 ~ 0.72) | <0.001 |  | 0.57 (0.44 ~ 0.72) | <0.001 |  | 0.58 (0.46 ~ 0.74) | <0.001 |
| *P* for trend |  | <0.001 |  |  | <0.001 |  |  | <0.001 |
| LDL-C | 1.49 (1.32 ~ 1.68) | <0.001 |  | 1.43 (1.26 ~ 1.62) | <0.001 |  | 1.39 (1.22 ~ 1.57) | <0.001 |
| LDL-C (Quartile) |  |  |  |  |  |  |  |  |
| 1 | 1.00 (Reference) |  |  | 1.00 (Reference) |  |  | 1.00 (Reference) |  |
| 2 | 1.11 (0.84 ~ 1.45) | 0.469 |  | 1.13 (0.86 ~ 1.48) | 0.395 |  | 1.12 (0.85 ~ 1.47) | 0.426 |
| 3 | 1.35 (1.04 ~ 1.75) | 0.023 |  | 1.33 (1.02 ~ 1.73) | 0.032 |  | 1.32 (1.02 ~ 1.72) | 0.037 |
| 4 | 1.91 (1.50 ~ 2.44) | <0.001 |  | 1.81 (1.41 ~ 2.32) | <0.001 |  | 1.74 (1.36 ~ 2.23) | <0.001 |
| *P* for trend |  | <0.001 |  |  | <0.001 |  |  | <0.001 |
| VLDL-C | 2.50 (1.57 ~ 3.98) | <0.001 |  | 1.91 (1.26 ~ 2.91) | 0.002 |  | 1.86 (1.25 ~ 2.77) | 0.002 |
| VLDL-C (Quartile) |  |  |  |  |  |  |  |  |
| 1 | 1.00 (Reference) |  |  | 1.00 (Reference) |  |  | 1.00 (Reference) |  |
| 2 | 1.03 (0.78 ~ 1.35) | 0.853 |  | 0.98 (0.75 ~ 1.29) | 0.897 |  | 0.98 (0.74 ~ 1.29) | 0.875 |
| 3 | 1.26 (0.98 ~ 1.62) | 0.071 |  | 1.20 (0.93 ~ 1.55) | 0.155 |  | 1.20 (0.93 ~ 1.54) | 0.171 |
| 4 | 1.71 (1.34 ~ 2.17) | <0.001 |  | 1.51 (1.18 ~ 1.92) | 0.001 |  | 1.48 (1.15 ~ 1.89) | 0.002 |
| *P* for trend |  | <0.001 |  |  | <0.001 |  |  | <0.001 |
| CRP | 1.04 (1.03 ~ 1.06) | <0.001 |  | 1.04 (1.03 ~ 1.05) | <0.001 |  | 1.05 (1.03 ~ 1.06) | <0.001 |
| CRP (Quartile) |  |  |  |  |  |  |  |  |
| 1 | 1.00 (Reference) |  |  | 1.00 (Reference) |  |  | 1.00 (Reference) |  |
| 2 | 1.23 (0.93 ~ 1.65) | 0.149 |  | 1.22 (0.92 ~ 1.63) | 0.172 |  | 1.27 (0.95 ~ 1.69) | 0.111 |
| 3 | 1.73 (1.32 ~ 2.27) | <0.001 |  | 1.67 (1.27 ~ 2.19) | <0.001 |  | 1.82 (1.38 ~ 2.40) | <0.001 |
| 4 | 2.51 (1.95 ~ 3.24) | <0.001 |  | 2.36 (1.82 ~ 3.06) | <0.001 |  | 2.70 (2.07 ~ 3.51) | <0.001 |
| *P* for trend |  | <0.001 |  |  | <0.001 |  |  | <0.001 |
| CO_2_ | 0.95 (0.92 ~ 0.98) | 0.002 |  | 0.96 (0.93 ~ 0.99) | 0.007 |  | 0.96 (0.93 ~ 0.99) | 0.021 |
| CO_2_ (Quartile) |  |  |  |  |  |  |  |  |
| 1 | 1.00 (Reference) |  |  | 1.00 (Reference) |  |  | 1.00 (Reference) |  |
| 2 | 0.91 (0.72 ~ 1.15) | 0.444 |  | 0.93 (0.73 ~ 1.18) | 0.542 |  | 0.93 (0.74 ~ 1.18) | 0.571 |
| 3 | 0.79 (0.62 ~ 0.99) | 0.049 |  | 0.81 (0.64 ~ 1.04) | 0.095 |  | 0.83 (0.65 ~ 1.05) | 0.124 |
| 4 | 0.78 (0.62 ~ 0.99) | 0.045 |  | 0.81 (0.63 ~ 1.03) | 0.084 |  | 0.85 (0.66 ~ 1.08) | 0.177 |
| *P* for trend |  | 0.025 |  |  | 0.053 |  |  | 0.121 |
| Ca | 3.95 (1.88 ~ 8.30) | <0.001 |  | 4.80 (2.26 ~ 10.20) | <0.001 |  | 4.03 (1.86 ~ 8.72) | <0.001 |
| Ca (Quartile) |  |  |  |  |  |  |  |  |
| 1 | 1.00 (Reference) |  |  | 1.00 (Reference) |  |  | 1.00 (Reference) |  |
| 2 | 1.01 (0.77 ~ 1.32) | 0.954 |  | 1.01 (0.77 ~ 1.33) | 0.936 |  | 0.95 (0.72 ~ 1.25) | 0.723 |
| 3 | 1.06 (0.82 ~ 1.35) | 0.668 |  | 1.08 (0.84 ~ 1.39) | 0.537 |  | 1.01 (0.78 ~ 1.30) | 0.954 |
| 4 | 1.50 (1.19 ~ 1.90) | <0.001 |  | 1.57 (1.23 ~ 1.99) | <0.001 |  | 1.44 (1.13 ~ 1.83) | 0.003 |
| *P* for trend |  | <0.001 |  |  | <0.001 |  |  | <0.001 |
| Mg | 0.07 (0.02 ~ 0.28) | <0.001 |  | 0.09 (0.02 ~ 0.33) | <0.001 |  | 0.07 (0.02 ~ 0.27) | <0.001 |
| Mg (Quartile) |  |  |  |  |  |  |  |  |
| 1 | 1.00 (Reference) |  |  | 1.00 (Reference) |  |  | 1.00 (Reference) |  |
| 2 | 0.67 (0.53 ~ 0.84) | <0.001 |  | 0.69 (0.54 ~ 0.87) | 0.002 |  | 0.68 (0.53 ~ 0.86) | 0.001 |
| 3 | 0.67 (0.53 ~ 0.85) | <0.001 |  | 0.66 (0.52 ~ 0.83) | <0.001 |  | 0.64 (0.51 ~ 0.81) | <0.001 |
| 4 | 0.59 (0.47 ~ 0.75) | <0.001 |  | 0.61 (0.48 ~ 0.78) | <0.001 |  | 0.59 (0.46 ~ 0.75) | <0.001 |
| *P* for trend |  | <0.001 |  |  | <0.001 |  |  | <0.001 |
| Cu | 1.04 (1.03 ~ 1.06) | <0.001 |  | 1.03 (1.01 ~ 1.04) | 0.003 |  | 1.03 (1.01 ~ 1.05) | 0.001 |
| Cu (Quartile) |  |  |  |  |  |  |  |  |
| 1 | 1.00 (Reference) |  |  | 1.00 (Reference) |  |  | 1.00 (Reference) |  |
| 2 | 0.96 (0.74 ~ 1.24) | 0.764 |  | 0.94 (0.72 ~ 1.22) | 0.629 |  | 0.96 (0.74 ~ 1.24) | 0.747 |
| 3 | 1.08 (0.84 ~ 1.39) | 0.534 |  | 1.01 (0.78 ~ 1.30) | 0.935 |  | 1.05 (0.81 ~ 1.36) | 0.703 |
| 4 | 1.53 (1.21 ~ 1.94) | <0.001 |  | 1.24 (0.97 ~ 1.58) | 0.084 |  | 1.28 (1.01 ~ 1.64) | 0.048 |
| *P* for trend |  | <0.001 |  |  | 0.051 |  |  | 0.027 |
| Fe | 0.98 (0.96 ~ 0.99) | <0.001 |  | 0.97 (0.96 ~ 0.99) | <0.001 |  | 0.97 (0.96 ~ 0.99) | <0.001 |
| Fe (Quartile) |  |  |  |  |  |  |  |  |
| 1 | 1.00 (Reference) |  |  | 1.00 (Reference) |  |  | 1.00 (Reference) |  |
| 2 | 0.64 (0.51 ~ 0.81) | <0.001 |  | 0.64 (0.50 ~ 0.81) | <0.001 |  | 0.63 (0.49 ~ 0.80) | <0.001 |
| 3 | 0.70 (0.56 ~ 0.89) | 0.003 |  | 0.68 (0.54 ~ 0.86) | 0.001 |  | 0.67 (0.53 ~ 0.85) | <0.001 |
| 4 | 0.67 (0.53 ~ 0.85) | <0.001 |  | 0.65 (0.51 ~ 0.82) | <0.001 |  | 0.65 (0.51 ~ 0.82) | <0.001 |
| *P* for trend |  | 0.001 |  |  | <0.001 |  |  | <0.001 |

Model 1: unadjusted model; Model 2: adjusted for age, BMI, plurality; Model 3: adjusted for age, BMI, plurality, parity, IVF conception.

PE, preeclampsia; OR, odds ratio; CI, confidence interval; PALB, prealbumin; GLB, globulin; ALT, alanine aminotransferase; ALP, alkaline phosphatase; GGT, gamma-glutamyltransferase; LDH, lactate dehydrogenase; CHE, cholinesterase; DBIL, direct bilirubin; UAR MoM, uric acid-albumin ratio multiple of the median; TyG MoM, triglyceride-glucose index multiple of the median; TC, total cholesterol; HDL-C, high-density lipoprotein cholesterol; LDL-C, low-density lipoprotein cholesterol; VLDL-C, very low-density lipoprotein cholesterol; CRP, c-reactive protein; CO_2_, total carbon dioxide; Ca, calcium; Mg, magnesium; Cu, copper; Fe, iron; BMI, body mass index; IVF, in vitro fertilization.

**Table S3. Threshold effects of laboratory markers on PE.**

| Variables | Outcome | OR (95%CI) | *P* |
| --- | --- | --- | --- |
| TBA (μmol/L) | Model 1 Fitting model by standard linear regression | 1.04 (1.00 ~ 1.09) | 0.079 |
|  | Model 2 Fitting model by two-piecewise linear regression |  |  |
|  | Inflection point | 2.591 |  |
|  | <2.591 | 1.24 (1.05 ~ 1.45) | 0.009 |
|  | ≥2.591 | 0.99 (0.89 ~ 1.11) | 0.911 |
|  | P for likelihood test |  | 0.015 |
| ALT (U/L) | Model 1 Fitting model by standard linear regression | 1.00 (1.00 ~ 1.01) | 0.008 |
|  | Model 2 Fitting model by two-piecewise linear regression |  |  |
|  | Inflection point | 37 |  |
|  | <37 | 1.03 (1.02 ~ 1.04) | <0.001 |
|  | ≥37 | 0.99 (0.99 ~ 1.00) | 0.278 |
|  | P for likelihood test |  | <0.001 |
| ALP (U/L) | Model 1 Fitting model by standard linear regression | 1.02 (1.02 ~ 1.03) | <0.001 |
|  | Model 2 Fitting model by two-piecewise linear regression |  |  |
|  | Inflection point | 41 |  |
|  | <41 | 0.96 (0.91 ~ 1.01) | 0.094 |
|  | ≥41 | 1.03 (1.02 ~ 1.03) | <0.001 |
|  | P for likelihood test |  | 0.007 |
| GGT (U/L) | Model 1 Fitting model by standard linear regression | 1.03 (1.02 ~ 1.03) | <0.001 |
|  | Model 2 Fitting model by two-piecewise linear regression |  |  |
|  | Inflection point | 35 |  |
|  | <35 | 1.06 (1.04 ~ 1.07) | <0.001 |
|  | ≥35 | 1.01 (0.99 ~ 1.02) | 0.338 |
|  | P for likelihood test |  | <0.001 |
| CHE (U/L) | Model 1 Fitting model by standard linear regression | 1.01 (1.01 ~ 1.01) | <0.001 |
|  | Model 2 Fitting model by two-piecewise linear regression |  |  |
|  | Inflection point | 253.962 |  |
|  | <253.962 | 1.00 (0.99 ~ 1.01) | 0.571 |
|  | ≥253.962 | 1.01 (1.01 ~ 1.01) | <0.001 |
|  | P for likelihood test |  | 0.009 |
| TyG MoM | Model 1 Fitting model by standard linear regression | 4.29 (3.40 ~ 5.42) | <0.001 |
|  | Model 2 Fitting model by two-piecewise linear regression |  |  |
|  | Inflection point | 0.83 |  |
|  | <0.83 | 0.65 (0.20 ~ 2.17) | 0.484 |
|  | ≥0.83 | 6.06 (4.50 ~ 8.17) | <0.001 |
|  | P for likelihood test |  | <0.001 |
| HDL-C (mmol/L) | Model 1 Fitting model by standard linear regression | 0.48 (0.37 ~ 0.63) | <0.001 |
|  | Model 2 Fitting model by two-piecewise linear regression |  |  |
|  | Inflection point | 1.84 |  |
|  | <1.84 | 0.30 (0.18 ~ 0.51) | <0.001 |
|  | ≥1.84 | 1.00 (0.51 ~ 1.98) | 0.997 |
|  | P for likelihood test |  | 0.027 |
| CRP (mg/L) | Model 1 Fitting model by standard linear regression | 1.05 (1.03 ~ 1.06) | <0.001 |
|  | Model 2 Fitting model by two-piecewise linear regression |  |  |
|  | Inflection point | 8.09 |  |
|  | <8.09 | 1.18 (1.13 ~ 1.24) | <0.001 |
|  | ≥8.09 | 1.01 (0.99 ~ 1.04) | 0.339 |
|  | P for likelihood test |  | <0.001 |
| Mg (mmol/L) | Model 1 Fitting model by standard linear regression | 0.07 (0.02 ~ 0.27) | <0.001 |
|  | Model 2 Fitting model by two-piecewise linear regression |  |  |
|  | Inflection point | 0.914 |  |
|  | <0.914 | 0.02 (0.00 ~ 0.13) | <0.001 |
|  | ≥0.914 | 1.27 (0.40 ~ 4.09) | 0.687 |
|  | P for likelihood test |  | <0.001 |
| Cu (μmol/L) | Model 1 Fitting model by standard linear regression | 1.03 (1.01 ~ 1.05) | 0.001 |
|  | Model 2 Fitting model by two-piecewise linear regression |  |  |
|  | Inflection point | 31.39 |  |
|  | <31.39 | 1.00 (0.97 ~ 1.03) | 0.825 |
|  | ≥31.39 | 1.09 (1.04 ~ 1.14) | <0.001 |
|  | P for likelihood test |  | 0.008 |
|  | Model 1 Fitting model by standard linear regression | 0.97 (0.96 ~ 0.99) | <0.001 |
| Fe (μmol/L) | Model 2 Fitting model by two-piecewise linear regression |  |  |
|  | Inflection point | 25.1 |  |
|  | <25.1 | 0.95 (0.93 ~ 0.97) | <0.001 |
|  | ≥25.1 | 1.04 (1.00 ~ 1.09) | 0.069 |
|  | P for likelihood test |  | 0.001 |

PE, preeclampsia; OR, odds ratio; CI, confidence interval; TBA, total bile acids; ALT, alanine aminotransferase; ALP, alkaline phosphatase; GGT, gamma-glutamyltransferase; CHE, cholinesterase; TyG MoM, triglyceride-glucose index multiple of the median; HDL-C, high-density lipoprotein cholesterol; CRP, c-reactive protein; Mg, magnesium; Cu, copper; Fe, iron.
